# Biomineralized Bimetallic Oxide Nanotheranostics for Multimodal Imaging-Guided Combination Therapy

**DOI:** 10.7150/thno.40715

**Published:** 2020-01-01

**Authors:** Jianrong Wu, Gareth R. Williams, Shiwei Niu, Yanbo Yang, Yu Li, Xuejing Zhang, Li-Min Zhu

**Affiliations:** 1 College of Chemistry, Chemical Engineering and Biotechnology, Donghua University, Shanghai 201620, P.R. China; 2 UCL School of Pharmacy, University College London, 29-39 Brunswick Square, London WC1N 1AX, UK

**Keywords:** iridium oxide/manganese dioxide, multimodal imaging, photothermal/photodynamic therapy, biomineralization, nanomedicine

## Abstract

The hypoxia of the tumor microenvironment (TME) often hinders the effectiveness of cancer treatments, especially O_2_-dependent photodynamic therapy (PDT).

**Methods:** An integrated iridium oxide (IrO_2_)-manganese dioxide (MnO_2_) nanotheranostic agent was fabricated through bovine serum albumin (BSA)-based biomineralization of Ir^3+^ and Mn^2+^. BSA was first covalently modified with chlorin e6 (Ce6), and used to fabricate multifunctional BSA-Ce6@IrO_2_/MnO_2_ nanoparticles (NPs) for computed X-ray tomography (CT) and photoacoustic (PA) imaging-guided PDT and photothermal (PTT) therapy of cancer. Extensive *in vitro* and *in vivo* studies were performed.

**Results:** The theranostic agent produced can relieve tumor hypoxia by the decomposition of endogenous H_2_O_2_ in cancer cells to oxygen. The oxygen generated can be exploited for improved PDT. Paramagnetic Mn^2+^ released from the NPs in the acidic TME permits magnetic resonance imaging (MRI) to be performed. The exceptional photothermal conversion efficiency (65.3%) and high X-ray absorption coefficient of IrO_2_ further endow the NPs with the ability to be used in computed CT and PA imaging. Extensive antitumor studies demonstrated that the BSA-Ce6@IrO_2_/MnO_2_ nanoplatform inhibits cancer cell growth, particularly after combined PTT and PDT. Systematic *in vivo* biosafety evaluations confirmed the high biocompatibility of the nanoplatform.

**Conclusion:** This work not only provides a novel strategy for designing albumin-based nanohybrids for theranostic applications but also provides a facile approach for extending the biomedical applications of iridium-based materials.

## Introduction

Cancer is a major global health problem, and among the leading causes of death worldwide 1-2. Despite enormous efforts made by researchers, there remain significant challenges because of the heterogeneity, diversity, and complexity of the tumor microenvironment (TME) 3-5. Hence, new technologies for early diagnosis, monitoring and therapy are much sought after. In recent years, various types of nanostructured materials have been explored for cancer theranostics due to their ability to target tumors and integrate diagnostic and imaging components (so-called “theranostics”) 6-9. Imaging technologies such as X-ray computed tomography (CT), near-infrared fluorescence (NIRF), magnetic resonance (MR), and photoacoustic (PA) modalities can be combined with chemotherapy, radiotherapy, or phototherapy into a single nanoscale platform to improve diagnostic or/and therapeutic efficacy 10-13.

The TME is typified by having a slightly acidic pH, and also being hypoxic as a result of deficient blood flow and insufficient oxygen supply 14. This not only induces tumor angiogenesis and metastasis, but also results in limited therapeutic outcomes in many cases. This issue is particularly acute for oxygen-dependent treatments such as photodynamic therapy (PDT) and radiotherapy 15-17. To overcome this problem, routes to deliver oxygen to the tumor regions have been explored 18-20. There is however a risk with such approaches, in that co-delivering oxygen with a chemotherapy or PDT could increase side effects caused by the generation of reactive oxygen species (ROS) 21. Therefore, a biosafety TME-responsive platform is required, the activity of which can be monitored and modulated. A wide variety of nanoscale structures have been developed to fulfill these needs 22-24. Among these, protein-based carriers have attracted particular attention owing to their inherent biocompatibility 25. For instance, albumin, the most abundant serum protein, has been shown to act as a versatile carrier for many cargos (chemotherapeutics, photosensitizers, and other hydrophobic molecules) since it is neither toxic nor immunogenic 26. Abraxane, based on albumin, is a clinically used nanomedicine which is potent in the treatment of several different types of cancer 27. The 3D structure of albumin provides amphiphilic properties, and there are abundant functional groups which can be exploited to construct multifunctional nanoplatforms 28.

Albumin has also been extensively researched as a nanoreactor to expropriate metal ions from solution and create protein coated metal oxide nanoclusters through biomineralization 29-31. For instance, Wang et al. successfully synthesized cypate-modified gadolinium oxide using albumin as the template and explored this in trimodal imaging-guided photothermal therapy 32. In another example, Yang et al. established a straightforward method to obtain a biocompatible Gd-integrated CuS nanoplatform for cancer theranostics *in vivo* 33. In addition, manganese dioxide (MnO_2_)-based nanostructures can be formed by using albumin as a template 34. There is a great deal of research that shows that MnO_2_ possesses high reactivity in the decomposition of endogenous H_2_O_2_ to produce O_2_; thus, it has attracted considerable interest for applications in O_2_-evolving PDT 35, 36. It should be noted that inflammation could be induced, however: if the MnO_2_ is retained in the body for a prolonged period of time then the local level of ROS could gradually increase 34. Minimizing H_2_O_2_-induced reactive oxygen inflammation during PDT is required to avoid undesirable side effects.

Beyond Mn, iridium (Ir) also has great potential in nanomedicine (in addition to numerous other applications) 37. Ir-based complexes are extremely efficient catalysts for oxygen evolution reactions 38. A recent study found that iridium oxide (IrO_2_) gave excellent contrast in both computed tomography and photoacoustic imaging 21. However, current research on Ir in nanomedicine is mainly focused on cyclometalated Ir(III) chemotherapeutic agents, and there is little literature on the application of IrO_2_ for *in vivo* theranostics. More importantly, there is to date no literature reports on the combination of IrO_2_ and MnO_2_ in nanomaterials for theranostic applications.

**Figure 1:**
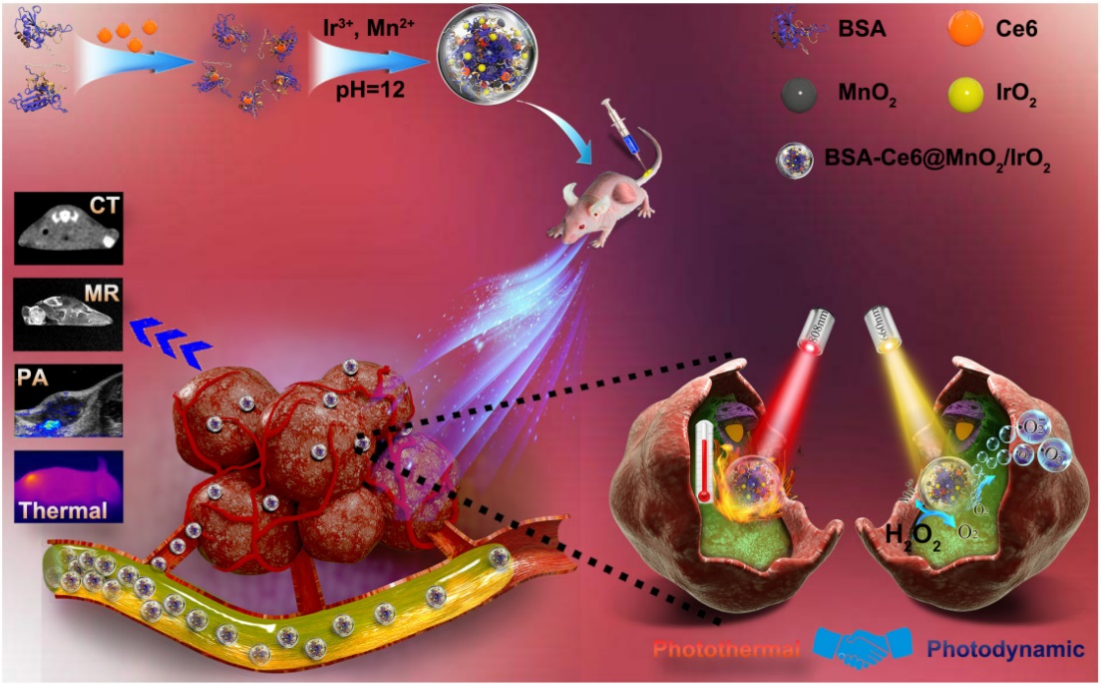
Schematic illustration of the concept behind using BSA-Ce6@IrO_2_/MnO_2_ for multiple bioimaging-guided tumor photothermal-photodynamic therapy.

Here, we report a versatile nanotheranostic agent generated through a bovine serum albumin (BSA)-based biomineralization process, sequestering both Ir^3+^ and Mn^2+^ ions (Figure 1). Chlorin e6 (Ce6), a hydrophobic photosensitizer commonly used in PDT, was first conjugated to BSA via the formation of an amide bond. Biomineralization of BSA-Ce6@IrO_2_/MnO_2_ was formed by the adsorption of Ir^3+^ and Mn^2+^ ions to BSA through the affinity of the carboxyl and amino groups of BSA toward metal ions, and then triggered by adjusting the pH value with NaOH. This was then exploited as a template for the synthesis of IrO_2_ and MnO_2_, yielding the composite BSA-Ce6@IrO_2_/MnO_2_. Owing to the presence of IrO_2_, BSA-Ce6@IrO_2_/MnO_2_ has high photothermal conversion efficiency and is suitable for CT imaging. The introduction of MnO_2_ can endow the nanoparticles with high reactivity in the decomposition of endogenous H_2_O_2_ to produce O_2_, which can overcome the hypoxia of the TME and thus enhance the efficacy of PDT. Meanwhile, Mn^2+^ ions released from the composite can act as a contrast agent for MR imaging 35. Furthermore, the catalase (CAT)-like activity of BSA-Ce6@IrO_2_/MnO_2_ enabled the system to reduce H_2_O_2_-related inflammation and protect healthy cells. Overall, we find that this novel nanomaterial holds great potential for cancer nanotheranostics and other oxygen-dependent treatments.

## Materials and Methods

### Materials

Bovine serum albumin (BSA), iridium trichloride (IrCl_3_), manganese chloride (MnCl_2_), N-(3-dimethylaminopropyl)-N-ethylcarbodiimide hydrochloride crystalline (EDC), 2,7-dichlorofluorescin diacetate (DCFH-DA), 1,3-diphenylisobenzofuran (DPBF), and N-hydroxysuccinimide (NHS) were procured from the Aladdin Reagent Co. (Shanghai, China). [Ru(dpp)_3_]Cl_2_ (RDPP), calcein acetoxymethyl ester (Calcein AM), and propidium iodide (PI), were purchased from Sigma-Aldrich (St Louis, MO, USA). Ce6 was sourced from J&K Scientific Ltd. A cell counting kit-8 (CCK-8) and tumor necrosis factor-α assay kit were obtained from the Beyotime Institute of Biotechnology (Shanghai, China). Dulbecco's modified Eagle medium (DMEM), fetal bovine serum (FBS), penicillin-streptomycin solution, and 0.05% trypsin-EDTA were sourced from Thermo Scientific (Beijing, China). MDA-MB-231 and 4T1 cells (human breast cancer cell lines), PC3 (a human prostate cancer cell line) and L929 cells (healthy murine fibroblasts) were provided by KeyGEN Bio TECH Co., Ltd (Nanjing, China). Deionized (DI) water (>18.2 MΩ·cm) was used for all experiments. All chemicals were used without additional purification.

### Synthesis of BSA-Ce6@IrO_2_/MnO_2_

Ce6 conjugated BSA (BSA-Ce6) was synthesized according to the literature 34. 1.0 mL of Ce6 solution (5 mg/mL in DMSO) was mixed with EDC (1.5 mg) and NHS (0.9 mg) and stirred for 1 h in the dark. BSA (0.1 g) was dissolved in 5 mL of deionized water. The activated Ce6-NHS was added into the BSA solution and stirred overnight. Next, the BSA-Ce6 product was centrifuged and washed with ethanol three times, then dialyzed (MWCO = 10 kDa) against deionized water for 24 h to remove free Ce6. The product was freeze-dried for subsequent use.

BSA-Ce6@IrO_2_/MnO_2_ NPs were prepared through a biomineralization strategy in the presence of Ir^3+^ and Mn^2+^. 20 mg BSA-Ce6 was dissolved in 10 mL water, into which 1 mL of MnCl_2_ solution (50 mM) and 1 mL of IrCl_3_ solution (50 mM) was slowly added and the resultant mixture stirred for 1 h at room temperature. Subsequently, a NaOH solution (2.0 M, 0.6 mL) was introduced to adjust the pH value to 12, and a purple solution was immediately formed. The mixture was stirred for 3 h at 37 ^o^C to allow MnO_2_ growth and then heated to 80 °C for another 12 h, under vigorous stirring. The suspension obtained was dialyzed (MWCO = 8-14 kDa) against deionized water for 24 h to remove excess precursors, and the BSA-Ce6@IrO_2_/MnO_2_ product freeze-dried. BSA-Ce6@IrO_2_ and BSA-Ce6@MnO_2_ nanoparticles were prepared following the same method but using a solution of only MnCl_2_ or IrCl_3_ and different temperatures (37 °C for MnO_2_, and 80 °C for IrO_2_).

### Characterization

The morphology of the BSA-Ce6@IrO_2_/MnO_2_ nanoparticles was characterized using high-resolution transmission electron microscopy (HR-TEM, Talos F200S, FEI, Hillsborough, OR, USA) equipped with an energy dispersive spectroscopy (EDS) attachment. Fourier transform infrared (FT-IR) spectra were recorded on a IRPrestige-21 spectrometer (Shimadzu, Kyoto, Japan). X-ray photoelectron spectra (XPS) were collected with an EscaLab 250Xi electron spectrometer (ThermoFisher, Waltham, MA, USA). Powder X-ray diffraction (XRD) patterns were obtained on a D8 ADVANCE X-ray diffractometer (Bruker, Billerica, MA, USA) supplied with Cu Kα radiation (λ = 1.5418 Å) at 40 kV and 40 mA. UV-Vis-NIR absorbance spectra were recorded on a UV-1700 spectrophotometer (Shimadzu, Kyoto, Japan). Concentrations of Mn and Ir were detected by inductively coupled plasma optical emission spectroscopy (ICP-OES, Prodigy7, Leeman Laboratories, Hudson, NH, USA). Prior to measurements samples were digested in aqua regia. Dynamic light scattering (DLS) and zeta potential measurements were performed with a Zetasizer Nano-ZS (Malvern Instruments, Malvern, UK). Circular dichroism spectra were measured using a MOS-450 system (BioLogic, Seyssinet-Pariset, France).

### Measurement of photothermal performance

To evaluate photothermal effects, 0.5 mL of BSA-Ce6@IrO_2_/MnO_2_ NP suspensions in water with various Ir concentrations (0-6 mM) were added into a quartz cuvette and irradiated under an 808 nm laser (1.0 W cm^-2^) for 5 min. Experiments were also performed with a fixed Ir concentration (3.0 mM) irradiated with different laser power densities for 5 min. Real-time temperature changes and thermal imaging were monitored by an infrared imaging camera (FLIR A300, LA, USA). The thermal stability and photothermal conversion efficiency were evaluated according to a previous study 24, and a suspension of BSA-Ce6@IrO_2_/MnO_2_ NPs (5 mM with respect to Ir) was irradiated for 5 min per cycle over five on-off cycles.

### Catalase (CAT)-like activity assay

To measure the CAT-like performance of BSA-Ce6@IrO_2_/MnO_2_, 5.0 mg of the NPs were dispersed in 20 mL of PBS (pH 6.0) and 250 µL of 0.2 mM H_2_O_2_ was added. After being stirred at room temperature for 10 min, O_2_ generation was measured with a JPBJ-608 dissolved oxygen meter (Shanghai REX Instrument Factory, Shanghai, China).

### Extracellular O_2_ generation

BSA-Ce6 or BSA-Ce6@IrO_2_/MnO_2_ was first dispersed in PBS (pH 5.5, 50 µg mL^-1^). 50 µL of RDPP solution (10 × 10^-3^ M in ethanol) was introduced and the mixture transferred into a cuvette, followed by the addition of 250 µL of 0.2 mM H_2_O_2_. At given time points, the fluorescence intensity of RDPP was recorded at an emission wavelength of 615 nm (FLS920 instrument, Edinburgh Instruments, Edinburgh, UK).

### Detection of singlet oxygen generation

10 µL of DPBF solution (in ethanol, 10 × 10^-3^ M) was added to 990 µL of BSA-Ce6@IrO_2_/MnO_2_ PBS suspensions (pH 6.0, [Ce6] = 1 µM) containing different concentrations of H_2_O_2_ (0, 0.1 and 0.2 mM) in a cuvette and mixed thoroughly. Next, the mixture irradiated with a 660 nm light (5 mW/cm^2^) for 10 min. A sample without laser irradiation was used as the control.

### CT/MR/PA imaging performance

For CT imaging *in vitro*, BSA-Ce6@IrO_2_/MnO_2_ NPs were dispersed in deionized water at different concentrations of Ir (0, 0.38, 0.75, 1.5, 3.0, 6.0, 12, and 25 mM) and then measured using a Philips 256-slice CT imaging system (Philips Medical Systems, Andover, MA, USA). CT images and Hounsfield unit (HU) values for each sample were captured. Clinical iobitridol was employed as a control. The imaging parameters were set according to a previous study (100 kV, 80 mA, and a slice thickness of 0.625 mm) 21.

For *in vitro* MR imaging, a 0.5 T NMI20 analyzing and imaging system (Shanghai NIUMAG Corporation, Shanghai, China) was employed. Before imaging, the Mn concentration released from BSA-Ce6@IrO_2_/MnO_2_ in different PBS buffers (pH 5.0 and 7.4) was determined by ICP-AES. *T_1_*-weighted MR images and the *T_1_*relaxation times of BSA-Ce6@IrO_2_/MnO_2_ suspensions in the different PBS buffers (pH 5.0 and 7.4), with or without treatments with H_2_O_2_ (0.2 mM), were recorded at different Mn concentrations. The parameters were the same as used in a previous study 35. The *r_1_* relaxivity was calculated through curve fitting the 1/*T_1_* relaxation time as a function of Mn concentration.

For *in vitro* PA imaging, BSA-Ce6@IrO_2_/MnO_2_ dispersions with different concentrations (0, 0.032, 0.16, 0.8. 4, 6.5 mM of Ir) were prepared, and PA images measured with the in Vision128 PA equipment (iThera Medical Inc., Munich, Germany) with excitation at 808 nm and a laser power of 1.6 mJ cm^-2^.

### Cell experiments

4T1 cells, MDA-MB-231 cells, PC3 cells, and L929 cells were cultured in RPMI-1640 (PC3) or DMEM (4T1, MDA-MB-231 and L929) medium supplemented with 10% v/v FBS and 1% v/v penicillin/ streptomycin solution. All cells were incubated at 37 °C in 5% CO_2_. The *in vitro* cytotoxicity of BSA-Ce6@IrO_2_/MnO_2_ was explored by seeding the different cell lines (5×10^3^ cells/well) in 96-well plates and then incubating at 37 ^o^C in 5% CO_2_ for 24 h. Subsequently, the culture medium was replaced with fresh medium containing different concentrations of BSA-Ce6@IrO_2_/MnO_2_. After incubation for another 24 h and washing with PBS, the cell viability was evaluated by the Cell Counting Kit-8 (CCK-8) assay.

The cellular uptake of BSA-Ce6@IrO_2_/MnO_2_ NPs was quantified using flow cytometry and ICP-OES. For flow cytometry, MDA-MB-231 cells were seeded in 12-well plates (5 × 10^4^ cells/well) and incubated with BSA-Ce6@IrO_2_/MnO_2_ or free Ce6 at equivalent Ce6 concentrations (2 µM) for 4 h, after which the cells were harvested and washed with PBS. The intracellular Ce6 fluorescence was determined by an Accuri®C6 flow cytometer (BD Biosciences, San Jose, CA, USA). For ICP-OES, the cell culture process was same as used for flow cytometry but with an incubation time of 6 h. After incubation, the cells were washed, trypsinized, centrifuged, and re-suspended in 1 mL of PBS before being digested in aqua regia for 24 h and diluted with water. The intracellular content of Ir and Mn was analyzed with ICP-OES as detailed above.

Intracellular ^1^O_2_ generation was detected with the DCFH-DA probe. MDA-MB-231 cells (2 × 10^5^ cells) were seeded in cell culture dishes and incubated at 37 ^o^C in 5% CO_2_ overnight, followed by the addition of DCFH-DA (1 mL, 10 µM in DMEM medium) and an additional 20 min incubation step. Next, the cells were treated with BSA-Ce6@IrO_2_, BSA-Ce6@MnO_2_, or BSA-Ce6@IrO_2_/MnO_2_ at a Ce6 concentration of 2 µM for another 24 h. After that, the cells were washed with PBS for three times and then exposed to 0.2 mM H_2_O_2_ in DMEM medium for another 45 min. The cells were finally washed with PBS and irradiated with 660 nm light (5 mW cm^-2^) for 15 min before being imaged with a TCS SP2 confocal laser florescence scanning microscope (Leica Microsystems, Mannheim, Germany) at an excitation wavelength of 488 nm. The production of the pro-inflammatory cytokine TNF-α was quantified with an enzyme-linked immunosorbent assay (ELISA) following the manufacturer's recommended protocol.

### *In vitro* phototherapy

MDA-MB-231 cells (5 × 10^4^ cells per well) were seeded in 96-well plates and incubated overnight at 37^ o^C in 5% CO_2_. The medium was replaced with 100 μL of fresh medium containing PBS and BSA-Ce6@IrO_2_/MnO_2_ at various Ce6 or Ir concentrations. After incubation for 24 h, the cells were washed with PBS three times. For PTT, the cells were next irradiated with an 808 nm laser (1.0 W cm^-2^) for 10 min. For PDT, the cells were exposed to a 660 nm LED light (5 mW cm^-2^) for 30 min. The temperature of the medium in the PDT experiments was maintained at ~4 °C to avoid any photothermal effects arising. For combination therapy, the cells were first irradiated with an 808 nm laser at 1.0 W cm^-2^ for 10 min, followed by irradiation by a 660 nm LED light at 5 mW cm^-2^ for 30 min. After irradiation, the cells were incubated for another 24 h. The demonstration of H_2_O_2_ enhanced phototherapy was realized through adding 0.2 mM H_2_O_2_ before illumination. The cell viabilities were then evaluated using the CCK-8 assay. The cytotoxicity of the BSA-Ce6@IrO_2_/MnO_2_ NPs was probed using an Axio Vert A1 fluorescence microscope (Carl Zeiss, Jena, Germany) after co-staining with Calcein AM and PI for 20 min. The synergistic effect of PTT/PDT was evaluated by combination index (CI) analysis 39, for which the CI was calculated as follows:

CI=D_1_/D_m1_+D_2_/D_m2_ (1)

Where D_1_ and D_2_ are the concentrations of Ce6 and Ir combined to produce a specified effect (50% reduction in cell viability). D_m1_ and D_m2_ are the doses of the single treatment required to obtain the same effect. Consequently, CI > 1 denotes antagonism; CI = 1 additivity, and CI < 1 synergism.

Flow cytometry experiments to assay apoptosis were also performed. MDA-MB-231 cells were seeded in 6-well plates (5 × 10^4^ cells per well in 500 μL of medium) and cultured overnight. The cells were next incubated with BSA-Ce6@IrO_2_/MnO_2_ ([Ir] = 3.0 mM, [Ce6] = 2 µM) for another 12 h with an 808 laser or 660 nm LED light irradiation treatment applied after 2 h incubation. Before laser irradiation, 0.2 mM H_2_O_2_ in DMEM medium was added. Following the 12 h period, the cells were rinsed thoroughly with PBS, and then treated with 0.5 mL trypsin. The harvested cells were suspended in PBS and centrifuged (2500 rpm, 5 min). Subsequently, they were co-stained with Annexin V-FITC and PI in binding buffer for 20 min in the dark. Flow cytometry was then performed on an Accuri®C6 flow cytometer (BD Biosciences, San Jose, CA, USA). The percentage of cells in each phase of apoptosis was quantified with the FCS Express Software (De Novo Software, Glendale, CA, USA).

### Animals and tumor model

Female Balb/c nude mice (~25 g) and Sprague-Dawley rats (~200 g) were purchased from Nanjing Peng Sheng Biological Technology Co., Ltd (Nanjing, China). All animal studies were performed following protocols approved by the Laboratory Animal Center of the Women's Hospital of Nanjing Medical University, the institutional committee for animal care, and the policy of the Chinese National Ministry of Health. To develop the tumor model, MDA-MB-231 cells (2 × 10^6^) in 100 μL of PBS were subcutaneously injected into the hind legs of each mouse. The tumor size was monitored with Vernier calipers every 2 days, and tumor volumes were calculated with the formula: V = [(length) × (width)^2^]/2. Body weight was also recorded. *In vivo* imaging and therapy experiments were conducted when the tumor size reached ca. 100 mm^3^.

### *In vivo* CT/MR/PA imaging

For CT and PA imaging *in vivo*, MDA-MB-231 tumor-bearing mice (n = 4) were anesthetized and intravenously injected with 100 μL of a BSA-Ce6@IrO_2_/MnO_2_ saline suspension prepared at an Ir concentration of 6 mM. At various time points after injection (0, 1, 2, 6, 12 and 24 h), images were acquired with a Philips 256-slice CT imaging system (Philips Medical Systems, Andover, MA, USA). The tumor CT values were also quantified. PA images were collected using the inVision128 PA equipment (iThera Medical Inc., Munich, Germany) at a wavelength of 808 nm (1.6 mJ cm^-2^).

For MRI experiments, MDA-MB-231 tumor-bearing nude mice were intravenously injected with a BSA-Ce6@IrO_2_/MnO_2_ saline suspension at a dose of 10 mg/kg (in terms of MnO_2_). *T_1_*-weighted MR images was obtained with a 3-T clinical MR scanner equipped with a special animal imaging coil (BioSpec 3T, Bruker Biospin, Billerica, MA, USA) prior to and at 2, 4 , and 12 h post injection.

### Pharmacokinetic study

Sprague-Dawley rats (n = 3) were intravenously injected with 200 μL of BSA-Ce6@IrO_2_/MnO_2_ NPs in saline (3.0 mM Ir). Approximately 20 µL of blood was collected at designated time intervals (0, 0.5, 1, 2, 3, 4, 6, 8, 12, 24, and 48 h), and then diluted with aqua regia. The Ir concentration was quantified with ICP-OES and the blood circulation half-life (t_1/2_) calculated with the following equations:


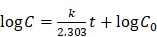
 (2)


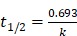
 (3)

Where C is the Ir concentration value at a given time, and C_0_ is the initial concentration in the blood.

### *In vivo* biodistribution, elimination and fluorescence imaging

For the biodistribution study, MDA-MB-231-bearing mice (n = 4) were intravenously injected with 200 μL of a suspension of BSA-Ce6@IrO2/MnO_2_ NPs in saline (3.0 mM Ir) at a dose of 10 mg/kg. The mice were sacrificed at different time points post-injection (2, 24, and 48 h) and the tumor tissue and major organs (liver, heart, lung, spleen, kidneys) were collected, weighed, and digested using aqua regia overnight. The Ir content of the samples was quantified using ICP-OES. To evaluate *in vivo* elimination, the feces of each mouse were collected, and each sample then dissolved in aqua regia. The amounts of Ir were analyzed through ICP-OES.

For *in vivo* fluorescence imaging, MDA-MB-231 tumor-bearing mice were randomly divided into three groups (n = 3) and given an intravenous injection of 200 μL of free Ce6 or BSA-Ce6@IrO_2_/MnO_2_ NPs (5 mg Ce6 equiv./kg) in saline via the tail vein. Fluorescence imaging was performed at different time intervals (1, 4, 8, 12, and 24 h post-injection) using a Lumina III *in vivo* imaging system (PerkinElmer, Waltham, MA, USA) with an excitation filter of 640 nm and an emission filter of 710 nm. 24 h after injection, the major organs (liver, spleen, kidney, heart, and lung) and tumor were excised for *ex vivo* imaging using the same instrument.

### *In vivo* PTT/PDT therapy

MDA-MB-231-bearing mice were randomly divided into five groups (n = 4): (1) saline (control group); (2) 808 nm + 660 nm laser (laser group); (3) BSA-Ce6@IrO_2_/MnO_2_ + 808 nm (PTT group); (4) BSA-Ce6@IrO_2_/MnO_2_ + 660 nm (PDT group); and, (5) BSA-Ce6@IrO_2_/MnO_2_ + 808 nm + 660 nm (combination group). The mice were intravenously injected with BSA-Ce6@IrO_2_/MnO_2_ NPs (Ce6 =5 mg kg^-1^, MnO_2_ = 10 mg kg^-1^, and IrO_2_ = 4.5 mg kg^-1^) in saline. At 12 h post-injection, the tumors in group 2, 3, and 5 were exposed to an 808 nm laser (1.0 W cm^-2^) for 10 min and the tumor temperature was recorded with an IR camera (A300, FLIR, LA, USA). Next, the tumors in group 2, 4, and 5 were irradiated with a 660 nm LED light (5 mW cm^-2^) for 30 min. The tumor size and body weight were measured every two days during the treatment period. After 15 days, the tumors were excised and weighed. The amount of TNF-α in the serum was measured with a standard enzyme-linked immunosorbent assay (ELISA, Cell Signaling Technology, Danvers, MA, USA) according to the manufacturer's instructions.

### Histology, histopathology and HIF-1α staining

After treatment, the excised tumors were fixed in paraformaldehyde and processed into paraffin, before being sliced to 4 µm thickness. The slices were stained with hematoxylin and eosin (H&E) or TdT-mediated dUTP nick-end labeling (TUNEL) for histological analysis, and with Ki-67 antigen for histopathology. HIF-1 α staining was also performed. The resultant slices were observed with a digital microscope (QWin, Microsystems, Mannheim, Germany). All staining experiments were conducted according to the manufacturer's guidelines.

### *In vivo* toxicity study and blood panel analysis

For *in vivo* toxicity evaluation, one mouse from each group was sacrificed and the main organs (hearts, livers, spleens, lungs, kidneys) were harvested for H&E staining. In addition, healthy female Balb/c mice (n = 4) were injected with 200 μL of BSA-Ce6@IrO_2_/MnO_2_ NPs (20 mg/kg) in saline through the tail vein. 0.5 mL of blood from each mouse was collected at different time points (1, 7, 28 days) for routine blood and biochemistry analysis.

### Statistical analysis

All results are reported as the mean ± S.D. and comparisons were performed using a two tailed Student's t test. All experiments, unless otherwise stated, were performed in triplicate. Statistical values are indicated according to the following: * P<0.05, ** P<0.01 and *** P<0.001.

## Results and discussion

### Preparation and characterization of BSA-Ce6@IrO_2_/MnO_2_

**Figure 2:**
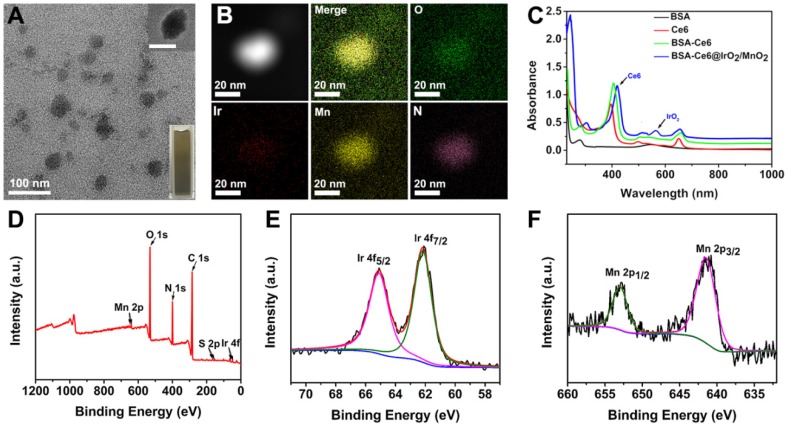
Characterizing data for the BSA-Ce6@IrO_2_/MnO_2_ nanoparticles; (A) TEM, HRTEM (inset) images and a photograph (inset); (B) HAADF-STEM images and EDX elemental mapping; (C) UV-Vis absorption spectra; and XPS data showing (D) the survey spectrum, (E) Ir 4f, and (F) Mn 2p spectra.

BSA-Ce6@IrO_2_/MnO_2_ NPs was prepared *via* a facile BSA-based biomineralization approach (Figure S1). Ce6 was firstly conjugated to BSA *via* the formation of amide bond, and the composite then acted to anchor both Ir^3+^ and Mn^2+^ ions owing to the strong affinity of the thiol and carboxyl groups of BSA for metal ions. After the formation of IrO_2_ and MnO_2_ nanocrystals coated with BSA, the nanotheranostic agent BSA-Ce6@IrO_2_/MnO_2_ was achieved (Figure 1 and S1). The contents of Ce6 in BSA-Ce6@IrO_2_/MnO_2_ was determined by UV-Vis absorbance spectroscopy and found to be 3.5%. As shown in Figure 2A, roughly spherical structures with an average size ca. 42 ± 3 nm are observed. The presence of metal-containing nanoparticles inside the overall structure is clear from the high-resolution TEM image (Figure 2A, inset). Elemental mapping revealed a homogeneous distribution of N, O, Ir and Mn, confirming the successful formation of IrO_2_ and MnO_2_ nanoparticles (Figure 2B) inside the BSA carrier. The presence of BSA was verified by FT-IR spectroscopy (Figure S2). The circular dichroism spectrum of BSA-Ce6@IrO_2_/MnO_2_ was essentially identical to that of free BSA, indicating the formation of the nanocomposite had no impact on the protein α-helix structure (Figure S3). The BSA-Ce6@IrO_2_/MnO_2_ NPs can be dispersed well in a range of media (Figures S4) with a hydrodynamic size of ca. 95 nm with a PDI of 0.16, and negative Zeta potential of -38.4 ± 3.4 mV in PBS. Due to the introduction of BSA coating shell, the NP dispersions in all three media are found to be stable during storage for at least one week, thus suggest the good colloidal stability of BSA-Ce6@IrO_2_/MnO_2_ NPs in physiological circumstances (Figure S4). UV-Vis-NIR absorption spectra are given in Figure 2C. Compared with the pure BSA spectrum, characteristic absorbance peak of Ce6 at 401 nm and 656 nm are observed for BSA-Ce6, confirming successful conjugation. Additional bands at 409 and 585 nm in the spectrum of BSA-Ce6@IrO_2_/MnO_2_ match well with those reported for Ce6 and IrO_2_ respectively, illustrating the presence of the IrO_2_ into the composite material. Survey XPS spectra (Figure 2D) further confirm the elemental composition of the materials. High-resolution spectra reveal Ir 4f_7/2_ and 4f_5/2_ peaks at 62.2 and 64.9 eV (Figure 2E) 21, and peaks at 653.1 and 641.4 eV from the Mn 2p_1/2_ and Mn 2p_3/2_ peaks of MnO_2_ (Figure 2F) 40. The O 1s spectra and C 1s spectra (Figure S5) both contain peaks from -C=O and M-O bonding. The XRD pattern of BSA-Ce6@IrO_2_/MnO_2_ exhibited characteristic Bragg reflections of IrO_2_ and MnO_2_ (Figure S6). All these observations together confirm the formation of MnO_2_ and IrO_2_.The Ir/Mn atomic ratios of BSA-Ce6@IrO_2_/MnO_2_ were analyzed using ICP-AES, and determined to be 5:3 for IrCl_3_/MnCl_2_ = 1/1.

### Photothermal properties and catalytic activity

**Figure 3:**
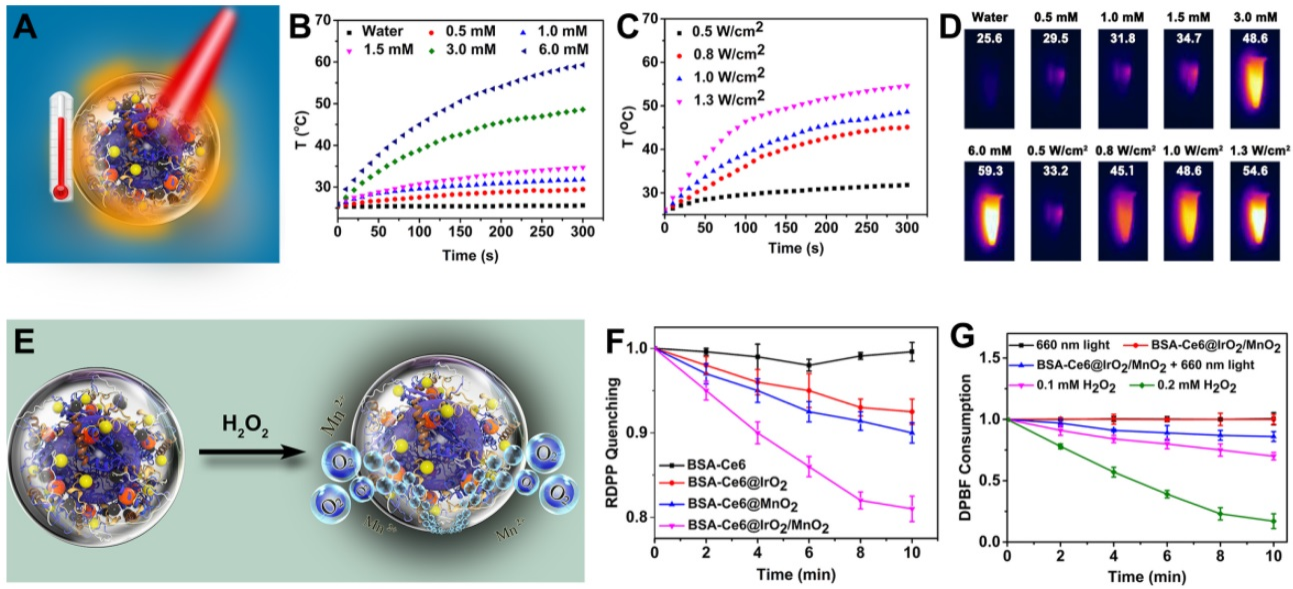
The application of BSA-Ce6@IrO_2_/MnO_2_ NPs as photothermal agents for the induction of local hyperthermia production and acidic H_2_O_2_-enhanced generation of O_2_ and ^1^O_2_. (A) A schematic illustration. (B) Heating curves of BSA-Ce6@IrO_2_/MnO_2_ dispersions at different Ir concentrations. (C) Heating curves at a fixed Ir concentration of 3.0 mM with different laser powers. (D) Photothermal images of BSA-Ce6@IrO_2_/MnO_2_ dispersions with different Ir concentrations irradiated for 5 min at varied power densities. (E) Schematic illustration of BSA-Ce6@IrO_2_/MnO_2_ generating O_2_ from H_2_O_2_. (F) The generation of oxygen determined by quenched RDPP fluorescence. (G) Consumption of DPBF triggered by BSA-Ce6@IrO_2_/MnO_2_, without or with 660 nm light irradiation and at different concentrations of H_2_O_2_ (0, 0.1 and 0.2 mM).

The photothermal properties of BSA-Ce6@IrO_2_/MnO_2_ were systematically investigated (Figure 3A). The temperature of an aqueous suspension of BSA-Ce6@IrO_2_/MnO_2_ increased sharply after 808 nm NIR irradiation (Figure 3B-D). The temperature of the dispersions (6 mM with respect to Ir) increases to 59.3 °C after laser (1.0 W/cm^2^) irradiation for 5 min, while pure water exhibits no significant temperature change (Figure 3B). Both concentration-dependent and laser-power-dependent photothermal effects are observed, indicating that hyperthermia generation can be tuned. Thermal images (Figure 3D) reveal clear changes in response to temperature increases, permitting easy visual monitoring. The photostability of BSA-Ce6@IrO_2_/MnO_2_ was measured and no change in performance is observed over five cycles of laser irradiation (Figure S7). The photothermal conversion efficiency of BSA-Ce6@IrO_2_/MnO_2_ was calculated to be 65.3% (Figure S8). This is considerably higher than many photothermal conversion agents reported in the literature, including black phosphorus quantum dots (28.4%) 41, Bi nanoparticles (45.3%) 42, boron nanosheets (42.5%) 43, and MoO_2_ (62.1%) 44. Thus, the BSA-Ce6@IrO_2_/MnO_2_ material can act as an effective agent for the photothermal ablation of a tumor.

It has been proven that IrO_2_ and MnO_2_ are able to act as catalysts for the decomposition of H_2_O_2_ into H_2_O and O_2_, similar to the endogenous catalase (CAT) enzyme 21, 35, 40. The high catalytic activity of IrO_2_ NPs for oxygen evolution reaction suggests the possible CAT-like activity of IrO_2_ NPs (Figure 3E). After incubation of H_2_O_2_ with BSA-Ce6@IrO_2_/MnO_2_ NPs for 5 min, oxygen generation could be observed by eye (Figure S9A). Clearly, rapid oxygen generation in the H_2_O_2_ solution was observed after BSA-Ce6@IrO_2_/MnO_2_ was added, while few amounts of oxygen bubbles were observed in the H_2_O_2_ solution after BSA-Ce6@IrO_2_ and BSA-Ce6@MnO_2_ were added. Also, BSA-Ce6 showed no catalytic activity to decompose H_2_O_2_. This can be proved by H_2_O_2_-triggered O_2_ production in a solution of BSA-Ce6@IrO_2_/MnO_2_, as detected by a portable dissolved-oxygen meter (Figure S9A).

The above results vividly illustrated the strong capability of BSA-Ce6@IrO_2_/MnO_2_ to induce decomposition of H_2_O_2_. This was further quantified using an RDPP O_2_ probe. The fluorescence intensity of RDPP descends rapidly after mixing H_2_O_2_ (0.2 mM) with BSA-Ce6@IrO_2_/MnO_2_ (Figure 3F), while BSA-Ce6 led to no change in intensity. Oxygen production triggered by BSA-Ce6@IrO_2_/MnO_2_ appeared to be faster than with analogous BSA-Ce6@IrO_2_ or BSA-Ce6@MnO_2_ nanoparticles containing only a single metal oxide, indicating the higher catalytic activity of BSA-Ce6@IrO_2_/MnO_2_. The DPBF chemical probe was employed to quantify ^1^O_2_ generation by BSA-Ce6@IrO_2_/MnO_2_ (Figure 3G). As expected, the absorption of DPBF was markedly decreased in the presence of BSA-Ce6@IrO_2_/MnO_2_, indicating ^1^O_2_ production. This effect becomes more significant at higher concentrations of H_2_O_2_. These results validate that the BSA-Ce6@IrO_2_/MnO_2_ can enhance the generation of ^1^O_2_ from H_2_O_2_. Therefore, the BSA-Ce6@IrO_2_/MnO_2_ NPs are expected to overcome the tumor hypoxia challenge and enhance PDT efficiency.

### *In vitro* imaging

**Figure 4:**
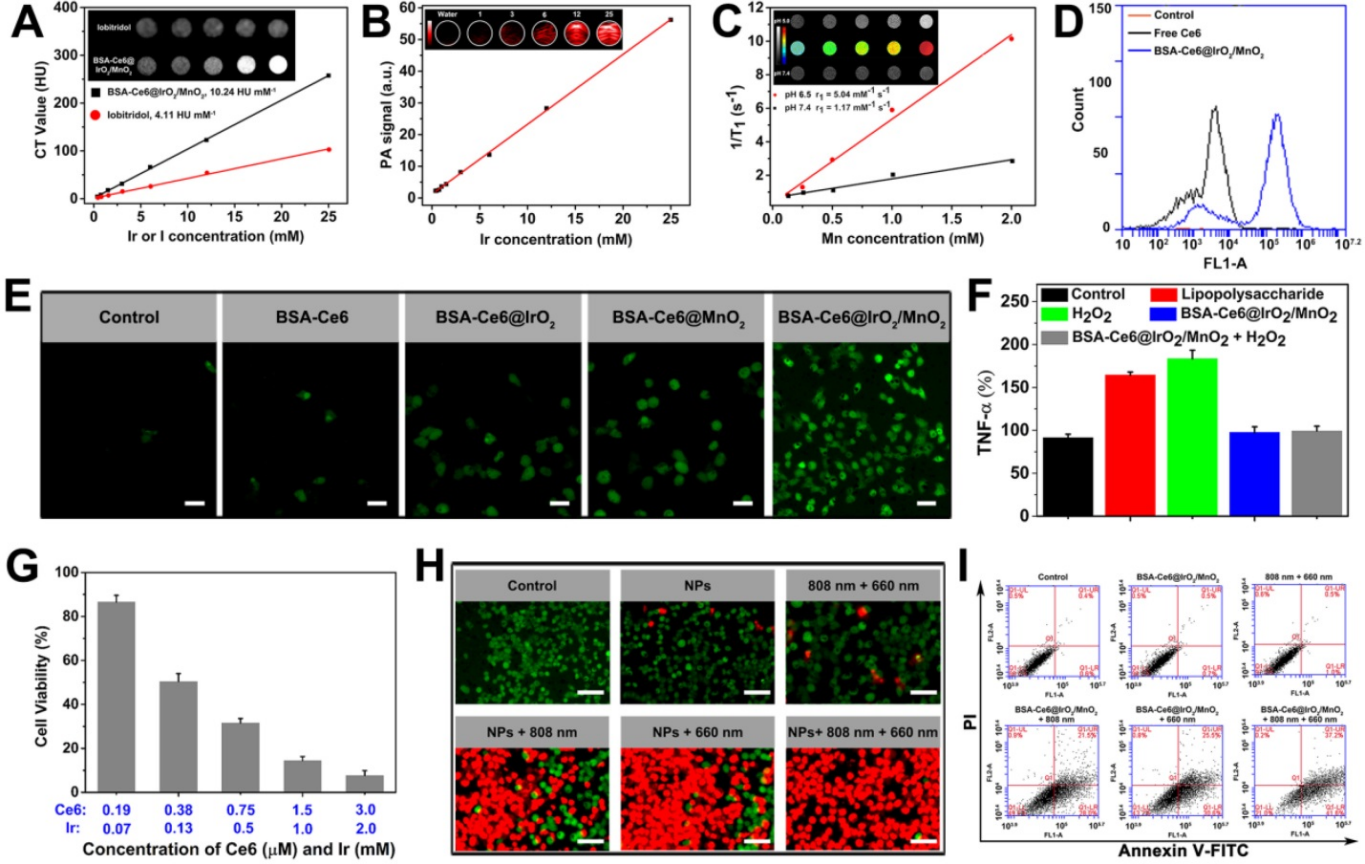
Imaging and cytotoxicity data obtained with BSA-Ce6@IrO_2_/MnO_2_ dispersions. (A) *In vitro* CT images and mean HU values. (B) PA images and signals. (C) T_1_-weighted MR images and relaxivity fits of BSA-Ce6@IrO_2_/MnO_2_ dispersions at pH 5.0 and 7.4 in the presence of H_2_O_2_. (D) Flow cytometry data obtained on of MDA-MB-231 cells incubated with Ce6, BSA-Ce6, and BSA-Ce6@IrO_2_/MnO_2_ NPs. (E) Intracellular ^1^O_2_ production after MDA-MB-231 cells were incubated with different formulations under laser irradiation (Ir: 3 mM, Mn: 2 mM). Scale bars: 50 µm. (F) Intracellular TNF-α level in L929 cell treated with LPS or BSA-Ce6@IrO_2_/MnO_2_. (G) Relative viabilities of MDA-MB-231 cells after incubation with BSA-Ce6@IrO_2_/MnO_2_ NPs and exposure to an 808 nm laser (1.0 W cm^-2^, 10 min) and 660 nm light irradiation (5 mW cm^-2^, 30 min). (H) Calcein-AM/PI staining images of MDA-MB-231 cells after different treatments (scale bar = 100 μm). (I) Flow cytometry results for Annexin V-FITC and PI co-stained MDA-MB-231 cells after different treatments.

The high atomic number (Z = 77) of Ir and high X-ray attenuation coefficient of IrO_2_ endows BSA-Ce6@IrO_2_/MnO_2_ with the ability to be employed in CT imaging. This was assessed by using clinical iobitridol as a control. As presented in Figure 4A, both the BSA-Ce6@IrO_2_/MnO_2_ NPs and iobitridol show concentration-dependent CT images with good linear correlation between Hounsfield units (HU) and concentration. The HU value of BSA-Ce6@IrO_2_/MnO_2_ was calculated to be 10.24 HU mM^-1^, which is significantly higher than that of iobitridol (4.11 HU mM^-1^). NIR absorption by IrO_2_ should also allow the BSA-Ce6@IrO_2_/MnO_2_ NPs to act as candidates for PA imaging. The PA signals and the brightness of the images rose in a linear fashion with increasing concentrations of NPs (Figure 4B). BSA-Ce6@IrO_2_/MnO_2_ thus has great potential for both CT and PA imaging.

MnO_2_ decomposes into Mn^2+^ and O_2_ in acidic environments. Mn release from BSA-Ce6@IrO_2_/MnO_2_ in different PBS buffers (pH 5.0 and 7.4) was determined by ICP-AES (Figure S10). It can be seen that Mn^2+^ was gradually released reaching a cumulative release of 76.3% at pH 5.0, but there is very little release at pH 7.4. These results confirmed that the BSA-Ce6@IrO_2_/MnO_2_ NPs have high sensitivity to acidic environments such as those typical of the TME. Mn^2+^ is highly paramagnetic, with 5 unpaired 3*d* electrons, and is potent as a *T_1_*-weighted contrast agent for MR imaging 45. *T_1_* relaxation data for BSA-Ce6@IrO_2_/MnO_2_ after incubation in different phosphate buffers (at pH 5.0 and 7.4) containing H_2_O_2_ are depicted in Figure 4C. An Mn^2+^ concentration-dependent increase in 1/*T_1_* was seen at pH 5.0, while much weaker signals were observed in the neutral buffer. The *r_1_* relaxivity of BSA-Ce6@IrO_2_/MnO_2_ at pH 5.0 is calculated to be 5.04 mM^-1^ s^-1^, notably higher than that at pH 7.4 (1.17 mM^-1^ s^-1^). The former is also greater than the clinically used Gd-based *T_1_* contrast, Magnevist (4.25 mM^-1^ s^-1^) 46. We also explored the *T_1_* relaxivity without any treatment with H_2_O_2_. The BSA-Ce6@IrO_2_/MnO_2_ NPs still provide MR contrast enhancement at pH 5 in a Mn concentration-dependent manner (Figure S11), and the signal intensity is only slightly lower than with H_2_O_2_ treatment at the same metal ion concentrations. This indicates that the presence of H_2_O_2_ did not materially affect the MRI contrast performance.

### *In vitro* biocompatibility, cellular uptake and photodynamic performance

The cytotoxicity of BSA-Ce6@IrO_2_/MnO_2_ was explored with a range of cell lines, including MDA-MB-231 and 4T1 cells, PC3 and L929 cells. Negligible toxicity was observed with all the cell types (Figure S12), even at high concentrations of BSA-Ce6@IrO_2_/MnO_2_ (250 µg/mL). Cellular uptake by MDA-MB-231 cells was probed via flow cytometry, and intracellular Ce6 fluorescence determined to be nearly 8-fold higher with cells exposed to BSA-Ce6@IrO_2_/MnO_2_ than with those given free Ce6 (Figure 4D and S13). Quantification of the cellular uptake of Ir and Mn using inductively coupled plasma-optical emission spectroscopy revealed that the BSA-Ce6@IrO_2_/MnO_2_ NPs exhibit time-dependent cellular uptake (Figure S14).

Intracellular O_2_ generation by BSA-Ce6@IrO_2_/MnO_2_ was examined by the RDPP O_2_ probe (Figure S15). The green fluorescence seen with untreated cells was almost completely quenched after treatment with BSA-Ce6@IrO_2_/MnO_2_, to a greater extent than with cells treated with BSA-Ce6@IrO_2_ or BSA-Ce6@MnO_2_. Such effective consumption of H_2_O_2_ by BSA-Ce6@IrO_2_/MnO_2_ NPs in MDA-MB-231 cells should effectively regulate the microenvironment of cancer cells. Next, intracellular ^1^O_2_ production by BSA-Ce6@IrO_2_/MnO_2_ was explored using 2',7'-dichlorodihydrofluorescein diacetate (DCFH- DA). After treatment with BSA-Ce6@IrO_2_/MnO_2_ under LED light irradiation (660 nm, 5 mW cm^-2^), the cells displayed significantly stronger green fluorescence, consistent with the generation of ^1^O_2_ (Figure 4E). Much weaker fluorescence and thus reduced ^1^O_2_ generation was found with either BSA-Ce6@IrO_2_ or BSA-Ce6@MnO_2_. These findings can be ascribed to increased degradation of H_2_O_2_ into O_2_ owing to the catalytic activity of IrO_2_ and MnO_2_ in BSA-Ce6@IrO_2_/MnO_2_. BSA-Ce6@IrO_2_/MnO_2_ can also act to reduce inflammation induced by H_2_O_2_ 47. While significant amounts of TNF-α were produced by L929 cells exposed to H_2_O_2_ or lipopolysaccharide (LPS) (Figure 4F), much lower levels were present if the cells were pretreated with BSA-Ce6@IrO_2_/MnO_2_.

### *In vitro* phototherapy efficacy

Further, *in vitro* therapeutic effects were investigated using the CCK-8 assay. MDA-MB-231 cells were incubated with various concentrations of BSA-Ce6@IrO_2_/MnO_2_, and then H_2_O_2_ (0.2 mM) was added to mimic the TME. The cells were treated by PTT (808 nm, 1.0 W cm^-2^, 10 min) alone, PDT (660 nm, 5 mW cm^-2^, 30 min) alone, and synergistic PTT and PDT using both conditions. A Ce6 dose-dependent toxicity was found in cells treated with PDT (Figure S16). The cytotoxicity of cells given PTT laser irradiation also rises with the concentration of NPs owing to their containing IrO_2_. When the cells were given combined PTT and PDT, over 90% of the cells was killed at the higher concentrations of Ir or Ce6 (Figure 4G). The combination index (CI) of PTT and PDT was calculated to be 0.32, confirming a potent synergistic effect. Live/dead cell staining assays (Figure 4H) confirm that almost complete cell death after simultaneously treatment with BSA-Ce6@IrO_2_/ MnO_2_ under 808 nm and 660 nm laser irradiation. A smaller number of dead cells were present after PTT or PDT alone. An apoptosis/necrosis assay was used to elucidate the mechanism underlying cell death (Figure 4I). Compared to cells treated with PBS, the apoptotic cell population (considering both early apoptotic and late-stage apoptotic cells) increases to 49.5% and 56.1% for cells treated with PTT alone and PDT alone, respectively. Upon a combined PTT and PDT treatment, the population of apoptotic cells increased to 78.8%. These results confirmed that the BSA-Ce6@IrO_2_/MnO_2_ NPs allow for simultaneous PDT and PTT.

### *In vivo* imaging

**Figure 5:**
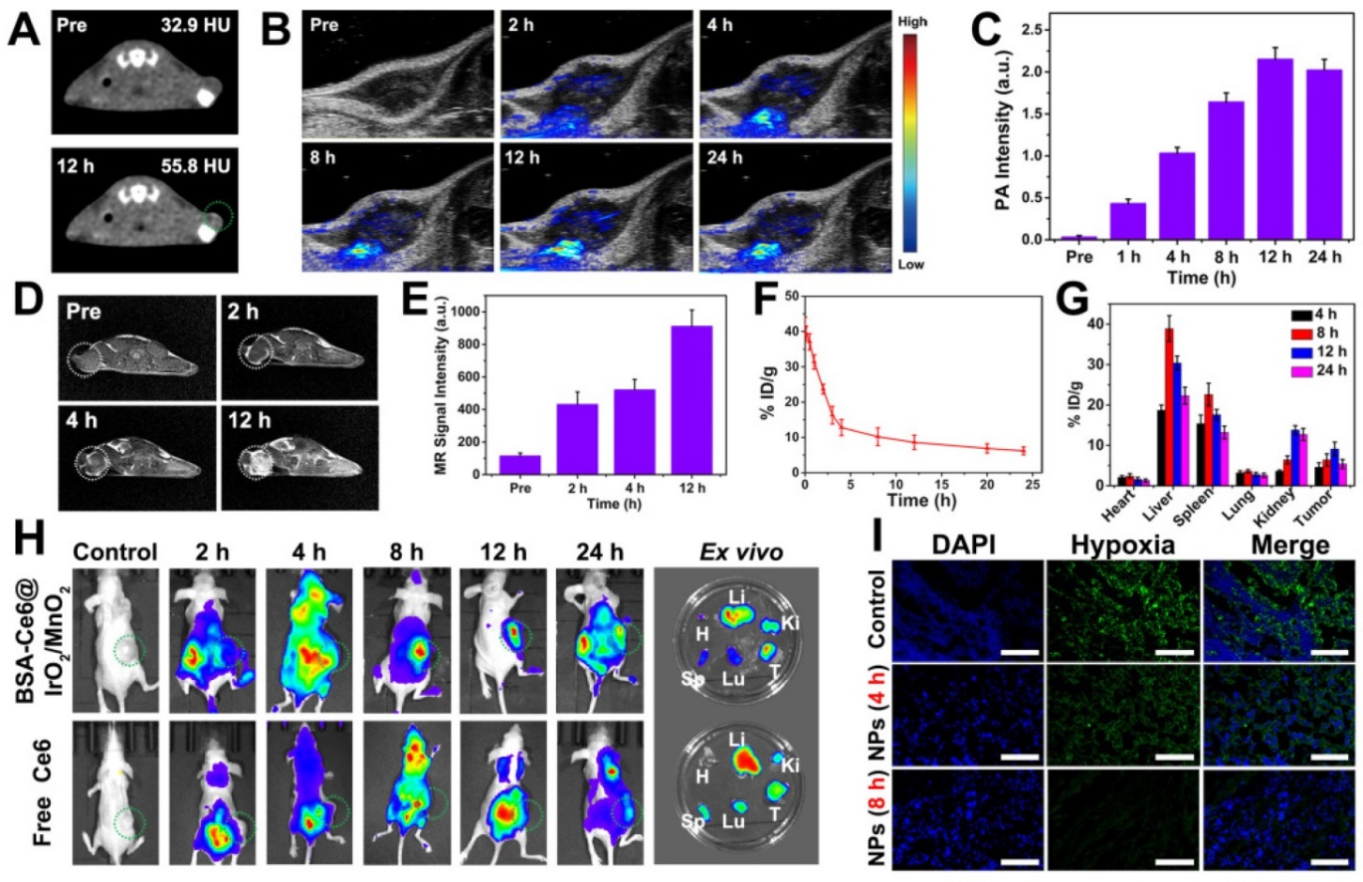
*In vivo* imaging, biodistribution, and up-regulation of oxygenation data obtained on MDA-MB-231 tumor bearing mice after i.v. injection of BSA-Ce6@IrO_2_/MnO_2_. (A) CT images. (B) PA images. (C) PA signal intensity as a function of time. (D) *In vivo T_1_*-weighted MR images. (E) MR signal intensities at different time points. (F) Blood circulation data quantified in terms of Ir concentrations in the blood. (G) The distribution of the NPs as a function of time, quantified in terms of Ir concentration. (H) *In vivo* fluorescence images taken at different time points and* ex vivo* images 24 h post-injection (H, Li, Sp, Lu, Ki, T denote the heart, liver, spleen, lung, kidney, and tumor respectively). (I) Representative immunofluorescence images of tumor slices collected from mice after the different treatments.

The potential of using the BSA-Ce6@IrO_2_/MnO_2_ as a theranositic nanoplatform *in vivo* was investigated using MDA-MB-231 tumor-bearing mice. CT images (Figure 5A) show clearly enhanced contrast at the tumor site, and the CT value reached 55.8 HU at 12 h after application of the NPs (Figure 5A). This is 1.7 times higher than that before injection (32.9 HU). The PA signals from the tumor region also increase in a time-dependent manner after injection, and reach a maximum value at 12 h (Figure 5B, C). A strong PA signal in the tumor is still observed after 24 h. *T_1_*-weighted MR imaging was also explored (Figure 5D and E). The tumor site turned brighter and exhibited gradual increases in the MR signal over time, reaching a maximum value 12 h after injection for 12 h. It is thus clear that the BSA-Ce6@IrO_2_/MnO_2_ system can be used for effective *in vivo* CT/PA/MR tri-modal imaging.

### Blood circulation and biodistribution

Nanoparticles of appropriate size are able to passively accumulate at a tumor site via the enhanced permeability and retention (EPR) effect 48, 49. The blood circulation profile of the BSA-Ce6@IrO_2_/MnO_2_ NPs was evaluated by determining the Ir concentration as a function of time. As shown in Figure 5F, the pharmacokinetics of BSA-Ce6@IrO_2_/ MnO_2_ follow a classical two-compartment model with a relatively long blood half-life (t_1/2α_=1.15 ± 0.85 h, t_1/2β_=12.41 ± 2.26 h). This should permit them to accumulate in the tumor via EPR. The *in vivo* distribution and biodegradability of BSA-Ce6@IrO_2_/ MnO_2_ after *i.v.* injection reveal that the tumor accumulation of Ir in mice exhibits a time-dependent pattern, peaking at 9.1% ID/g at 12 h post-inection (Figure 5G). The BSA-Ce6@IrO_2_/MnO_2_ nanoparticles also accumulate in the liver and kidney to some extent, due to non-specific uptake by reticuloendothelial cells 49. The Ir level present in all organs decreased gradually over time, while the amount in the feces (Figure S17) increased. This shows that BSA-Ce6@IrO_2_/MnO_2_ can be rapidly eliminated from the body via excretion in feces, as a result of their ultrasmall size and biodegradable nature. This should enable them to avoid potential toxicity concerns *in vivo*. These results suggested the fabricated nanoplatform that is purely composed by biocompatible and biodegradable components, with substantial potential for future clinical translation. As depicted in Figure 5H, Ce6 fluorescence was observed throughout the whole body 2 h after intravenous injection. With the NP formulation there is an obvious accumulation of Ce6 in the tumor with time. In contrast, minimal Ce6 fluorescence was detected in the tumor site in mice injected with free Ce6, owing to the rapid excretion of Ce6. *Ex vivo* fluorescence imaging performed on the major organs (heart, spleen, lung, and kidney) and tumor tissues (Figure 5H) confirmed the *in vivo* findings, and is also consistent with the Ir concentration data in Figure 5G. Mice treated with BSA-Ce6@IrO_2_/MnO_2_ exhibited strong Ce6 fluorescence and high Ir concentrations in the tumor.

### *In vivo* combined PTT-PDT treatment efficiency

**Figure 6:**
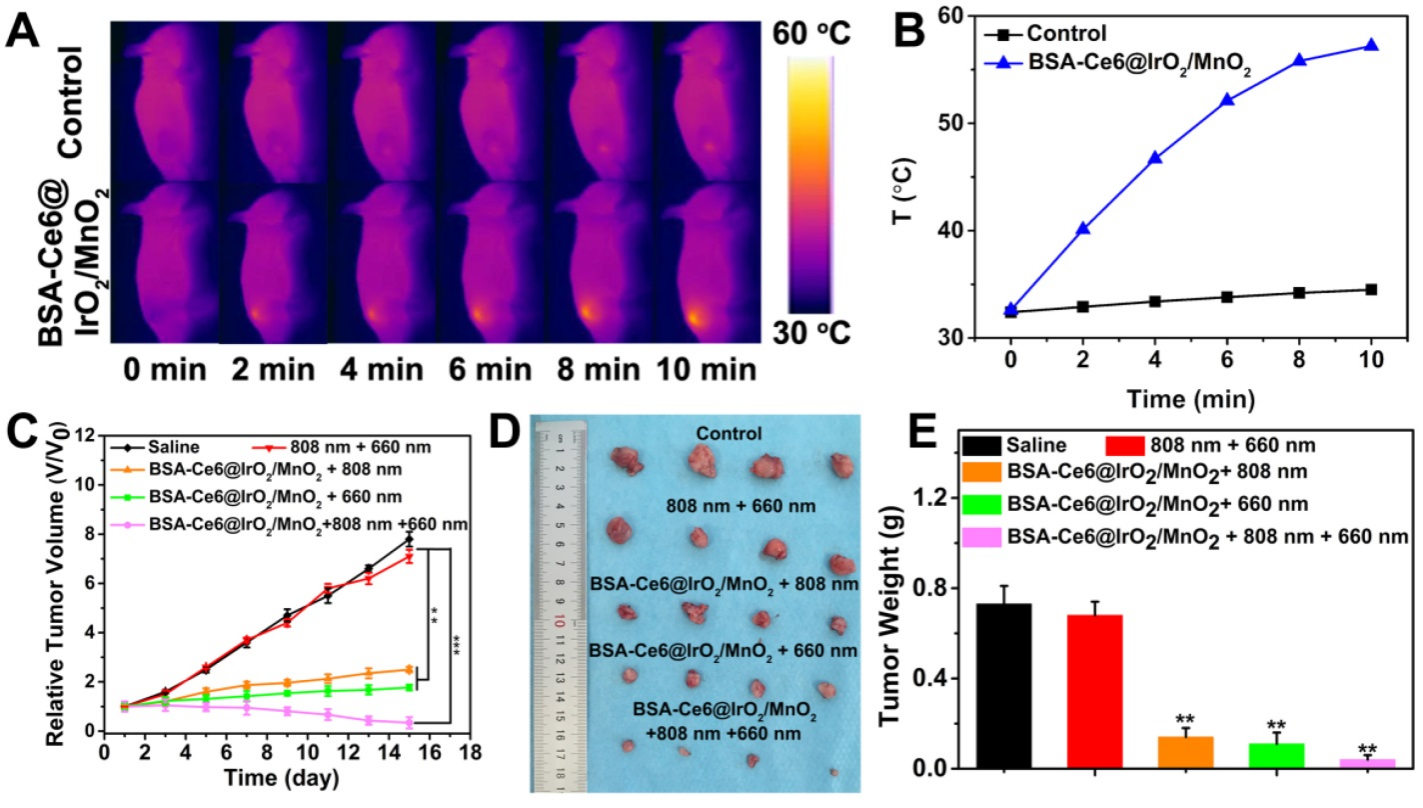
(A) *In vivo* thermal imaging and (B) time-dependent temperature changes of mice after treated with saline and BSA-Ce6@IrO_2_/MnO_2_ under an 808 nm laser irradiation for 10 min. (C) Relative tumor volume curves of MDA-MB-231 tumor bearing nude mice as a function of time and treatment. (D) Photographs and (E) weights of the tumors excised after 15 days of treatment. ***P < 0.001, **P < 0.01.

Next, the *in vivo* antitumor study was performed in the MDA-MB-231 tumor-bearing mouse model. Tumor slices extracted from mice were stained with an anti-pimonidazole antibody to permit hypoxia to be imaged by confocal microscopy (Figure 5I). Extensive green fluorescence was observed in mice treated with saline, indicating local hypoxia in the tumor tissue. In marked contrast, tumor slices from mice receiving BSA-Ce6@IrO_2_/MnO_2_ showed significantly reduced green fluorescence, particularly 8 h after injection. This demonstrates that BSA-Ce6@IrO_2_/MnO_2_ can relieve hypoxia in tumors by decomposing H_2_O_2_ to produce O_2_ *in vivo*, thus improving the efficacy of PDT. The thermal images (Figure 6A) show that the mice injected with BSA-Ce6@IrO_2_/MnO_2_ displayed distinct increases in the temperature at the tumor, which can reach 57.2 °C upon laser irradiation (Figure 6B). BSA-Ce6@IrO_2_/ MnO_2_ can thus act as an effective and photothermal agent for PTT. To verify this, MDA-MB-231-tumor bearing mice were randomly divided into five groups (n = 4 per group) and treated as follows: saline (control), 808 nm + 660 nm laser (laser group), BSA-Ce6@IrO_2_/MnO_2_ + 808 nm laser (PTT group), BSA-Ce6@IrO_2_/MnO_2_ + 660 nm laser (PDT group), and BSA-Ce6@IrO_2_/MnO_2_ + 808 nm + 660 nm laser (combination group). Compared to the control group and the laser group, the PTT and PDT groups showed distinct reductions in tumor volume (Figure 6C). The combination group gave the smallest tumor volume (V/V_0_ = 0.23), indicating a marked synergistic effect arising from combined PTT and PDT using BSA-Ce6@IrO_2_/MnO_2_. Digital photos and the average tumor weights of the excised tumors at the end of the treatment period (Figure 6D and E) clearly demonstrate that the mice treated with BSA-Ce6@IrO_2_/MnO_2_ plus 660 nm and808 nm irradiation have the smallest tumor size.

**Figure 7:**
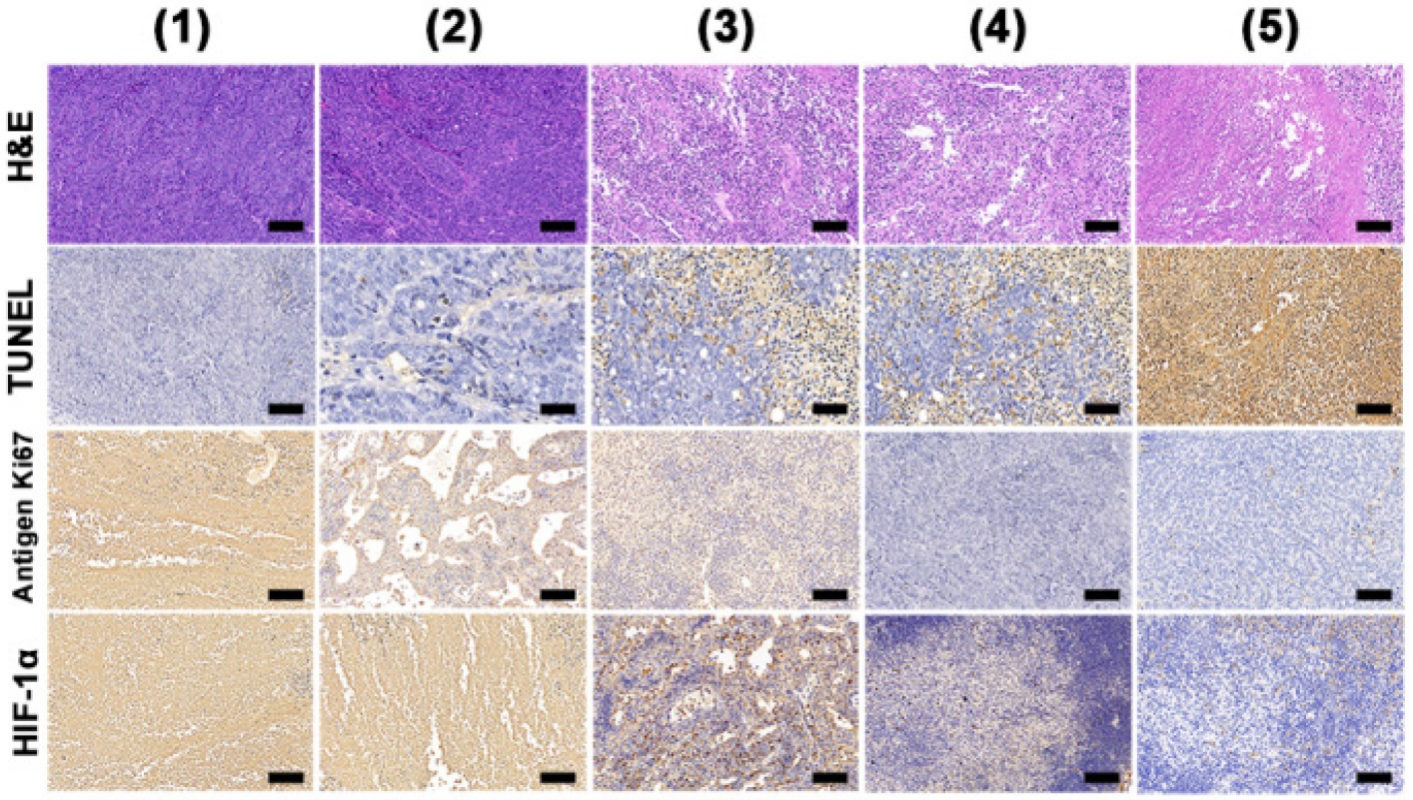
H&E, TUNEL, antigen Ki-67 immunohistochemistry and HIF-1α staining of tumor slides from MDA-MB-231 tumor bearing mice. Scale bars: 50 μm. Data were collected on day 15 from mice treated with (1) saline, (2) 808 nm + 660 nm laser irradiation, (3) BSA-Ce6@IrO_2_/MnO_2_ + 808 nm laser, (4) BSA-Ce6@IrO_2_/MnO_2_ + 660 nm laser, and (5) BSA-Ce6@IrO_2_/MnO_2_ + 808 nm + 660 nm lasers, respectively.

With all treatments, the mouse body weights exhibit no obvious change with time, implying there are minimal off-target side effects (Figure S18). To further explore the antitumor efficacy, hematoxylin and eosin (H&E) and terminal deoxynucleotidyl transferase dUTP-biotin nick end labeling (TUNEL) staining were performed. These reveal abundant tumor tissue necrosis in the combination group (Figure 7), and reduced extents of necrosis where mice were given PDT or PTT alone. Immunochemical staining of Ki-67 analysis showed much greater suppression of cell proliferation (brown-stained cells) in the combination group. A hypoxia inducible factor (HIF-1α) staining assay was also performed and it can be seen that the tumor tissues were largely stained blue in groups given BSA-Ce6@IrO_2_/MnO_2_ NPs, indicating relief of the hypoxia in the TME. Quantitative analyses of the H&E, TUNEL Ki67 and HIF-1α data are shown in Figure S19. The results confirmed the synergistic therapy acts to both inhibit cell proliferation and induce apoptosis in tumor tissue. Further, it can be seen that BSA-Ce6@IrO_2_/ MnO_2_ NPs induced significant HIF-1a downregulation in the tumor tissue. These results together confirmed the potent synergistic effects induced by using combined PDT and PTT therapy with the BSA-Ce6@IrO_2_/MnO_2_ formulation.

### *In vivo* biocompatibility and biosafety

The biosafety of BSA-Ce6@IrO_2_/MnO_2_ nanoplatform was evaluated by H&E-staining the major organs from the mice after 15 days (Figure S20). No obvious inflammation or damage to the major organs was observed in any case, suggesting minimal toxic side effects during therapy. Survival curves showed that mice treated with BSA-Ce6@IrO_2_/MnO_2_ in combination with 660 nm and 808 nm laser irradiation all survived over an experimental period of 48 days (Figure S21), while mice give either PDT or PTT alone lived for only 34-38 days. Serum biochemistry assays and complete blood panel tests revealed no statistical differences between the BSA-Ce6@IrO_2_/MnO_2_ group and mice treated with PBS in most hematochemistry and physiochemistry parameters (Figure S22) confirms that BSA-Ce6@IrO_2_/MnO_2_ has no significant renal or hepatic toxicity. The TNF-α concentration in the serum of mice after different treatments (Figure S23) was further investigated. An injection of 1 mM H_2_O_2_ induces elevated expression of TNF-α, but TNF-α levels are the same as the control with mice treated with BSA-Ce6@IrO_2_/MnO_2_, even if 1 mM H_2_O_2_ is also introduced. The NPs can thus inhibit inflammatory cytokines induced by H_2_O_2_ *in vivo*.

## Conclusions

In conclusion, we have successfully fabricated for the first time an albumin-biomineralized nanotheranostic for CT/MRI/PA trimodal imaging and combination PDT and PTT of tumors. This has been achieved by integrating IrO_2_ and MnO_2_ into a Ce6 conjugated-bovine serum albumin, yielding BSA-Ce6@IrO_2_/MnO_2_ nanoparticles with a uniform diameter of ca. 110 nm and good *in vitro* and *in vivo* biocompatibility. The large X-ray attenuation coefficient of Ir endows the BSA-Ce6@IrO_2_/MnO_2_ formulation with excellent CT contrast. The formulation also shows high NIR absorption, and can hence be used for PA and photothermal imaging, with strong photothermal conversion efficiency (65.3%). BSA-Ce6@IrO_2_/MnO_2_ further catalyzes H_2_O_2_ decomposition to generate O_2_, overcoming tumor hypoxia and further improving the efficacy of PDT. Mn^2+^ ions released from the nanoparticles in the acidic microenvironment of the tumor permit MRI imaging with a high *r_1_* relaxivity (5.04 mM^-1^ s^-1^). A systematic *in vitro* and *in vivo* evaluation confirmed highly effective synergistic therapy and excellent CT/MRI/ PA imaging capabilities of the BSA-Ce6@IrO_2_/MnO_2_ system. In the tumor, it induces extensive cell death, particularly when used for combined PDT/PTT. No damage is caused to heathy tissues, which are protected against inflammatory cytokines. This “all in one” system can act as a powerful nanotheranostic integrating photothermal therapy, photodynamic therapy, multimode imaging and catalytic treatment of hypoxia.

## Supplementary Material

Supplementary figures.

## Abbreviations

BSA: Bovine serum albumin
Calcein AM: calcein acetoxymethyl ester
CAT: Catalase
CCK-8: Cell Counting Kit-8
Ce6: chlorin e6
CI: combination index
CT: computed X-ray tomography
DCFH-DA: 2, 7-dichlorofluorescin diacetate
DLS: Dynamic light scattering
DMEM: Dulbecco's Modified Eagle Medium
DPBF: 1,3-diphenylisobenzofuran
ELISA: enzyme-linked immunosorbent assay
EPR: enhanced permeability and retention
H&E: hematoxylin and eosin
HIF-1α: hypoxia inducible factor-1α
ICP-OES: inductively coupled plasma optical emission spectroscopy
IrO_2_: iridium oxide
LPS: lipopolysaccharide
MnO_2_: manganese dioxide
MRI: magnetic resonance imaging
PA: photoacoustic
PDT: Photodynamic therapy
PTT: Photothermal therapy
ROS: reactive oxygen species
TEM: Transmission electron microscopy
TME: tumor microenvironment
TNF-α: tumor necrosis factor
TUNEL: TdT-mediated dUTP nick-end labeling
XPS: X-ray photoelectron spectra
