# Biomineralized Bimetallic Oxide Nanotheranostics for Multimodal Imaging-Guided Combination Therapy: Erratum

**DOI:** 10.7150/thno.78027

**Published:** 2022-11-02

**Authors:** Jianrong Wu, Gareth R. Williams, Shiwei Niu, Yanbo Yang, Yu Li, Xuejing Zhang, Li-Min Zhu

**Affiliations:** 1 College of Chemistry, Chemical Engineering and Biotechnology, Donghua University, Shanghai 201620, P.R. China; 2 UCL School of Pharmacy, University College London, 29-39 Brunswick Square, London WC1N 1AX, UK

In the original publication, in the results of flow cytometry analysis, Figure 4I showed the flow cytometry results for MDA-MB-231 cells after different treatments. There were confusion and error in the three groups of experiments due to our carelessness in sorting out the data. During the assembling of figure, we mistakenly put the same image of control, BSA-Ce6@IrO_2_/MnO_2_ and 808 nm + 660 nm group. The corrected Figure 4I is shown below. The authors confirm that the correction made in this erratum would not affect the result and conclusion of the published article. The authors sincerely apologize for any inconvenience that the errors may have caused.

## Figures and Tables

**Figure 4:**
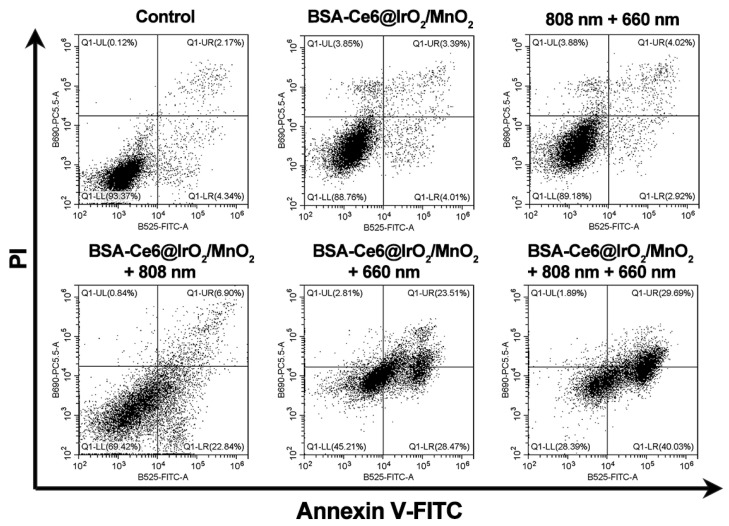
(I) Flow cytometry results for Annexin V-FITC and PI co-stained MDA-MB-231 cells after different treatments.

