# Supplementary material for: Biomineralized Bimetallic Oxide Nanotheranostics for Multimodal Imaging-Guided Combination Therapy
Source: Theranostics. 2020 Jan 1;10(2):841–55. doi: 10.7150/thno.40715 (PMC6929990; doi:10.7150/thno.40715)
Supplement: Supplementary file 1 — Supplementary figures. [file thnov10p0841s1.pdf]

# Supporting Information

## Biomineralized Bimetallic Oxide Nanotheranostics for Multimodal Imaging-Guided Combination Therapy

Jianrong Wu[1], Gareth R. Williams[2], Shiwei Niu[1], Yanbo Yang[1], Yu Li[1], Xuejing Zhang[1], Li-Min Zhu[1]✉

[1]College of Chemistry, Chemical Engineering and Biotechnology, Donghua University, Shanghai 201620, P.R. China

[2]UCL School of Pharmacy, University College London, 29-39 Brunswick Square, London WC1N 1AX, UK

✉ Corresponding author: Email addresses: lzhu@dhu.edu.cn (L.-M. Zhu).

# Supplementary Figures

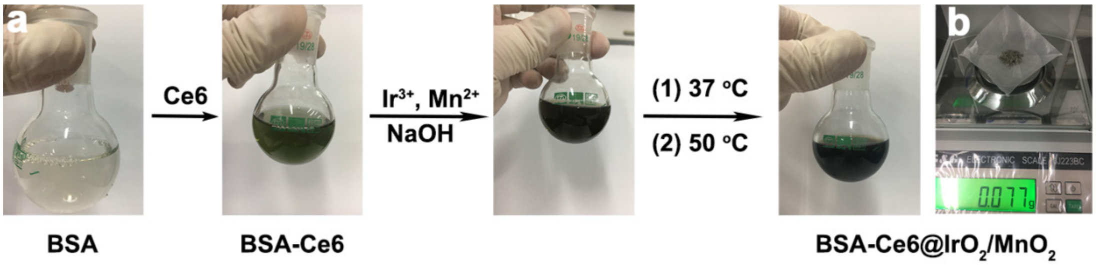


**Figure S1.** (a) The synthetic process for BSA-Ce6@$IrO_2$/$MnO_2$ nanoparticles. (b) A digital photo of the lyophilized BSA-Ce6@$IrO_2$/$MnO_2$ nanoparticles.

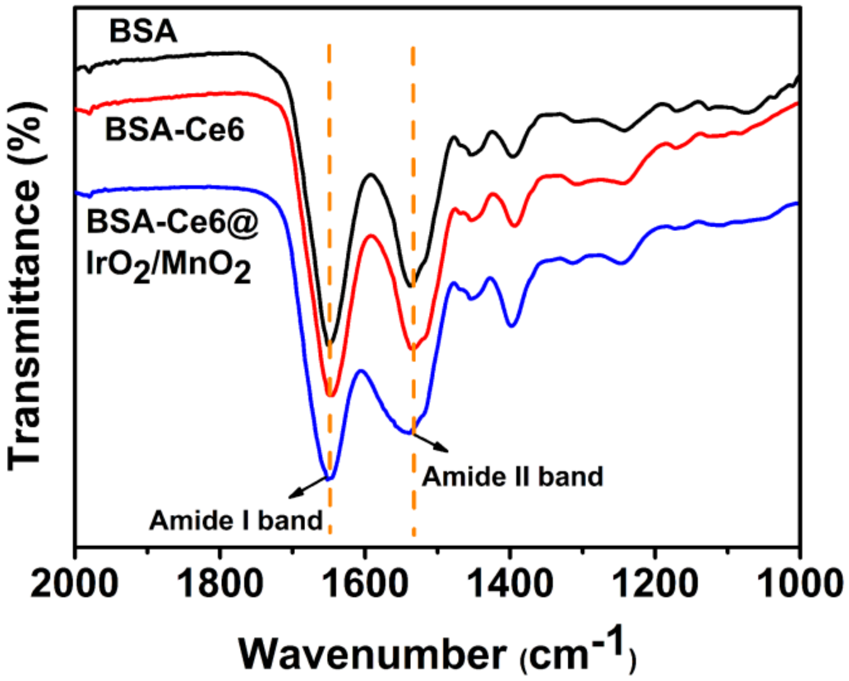


**Figure S2.** FT-IR spectra of BSA, BSA-Ce6, and BSA-Ce6@$IrO_2$/$MnO_2$.

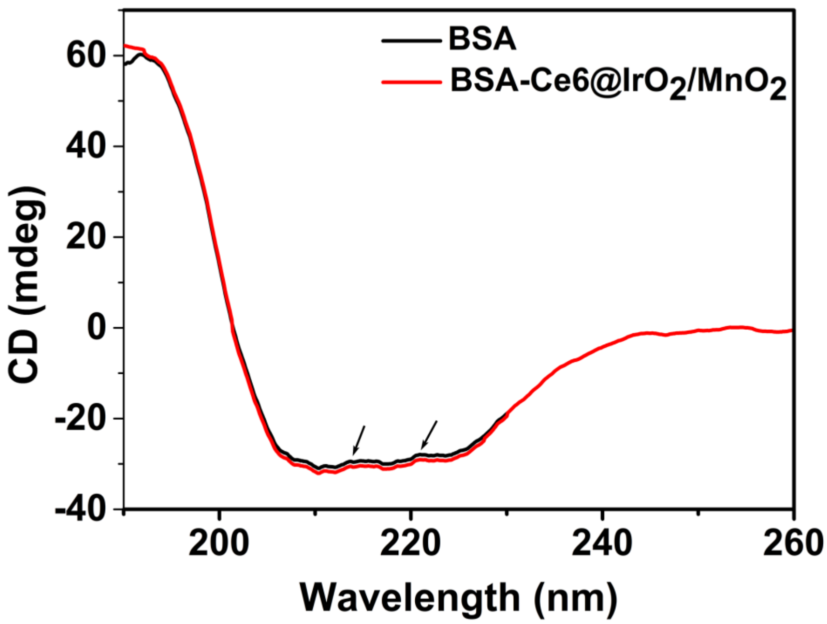


**Figure S3.** Circular dichroism spectra of BSA and BSA-Ce6@$IrO_2$/$MnO_2$.

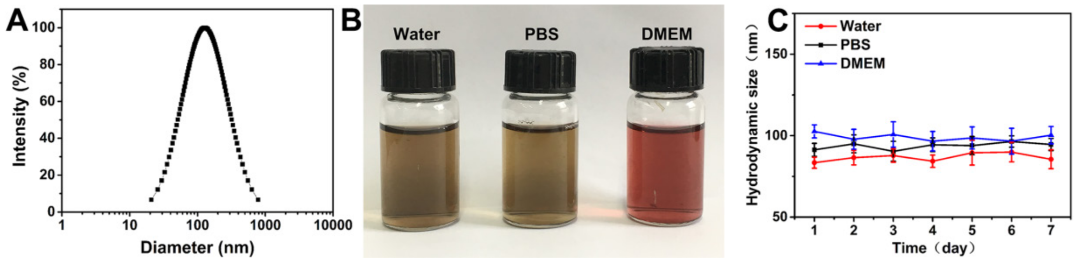


**Figure S4.** Data on BSA-Ce6@$IrO_2$/$MnO_2$ dispersions. (A) The hydrodynamic diameter in PBS. (B) Photographs of the dispersions in water, PBS, and DMEM. (C) The hydrodynamic sizes of the NPs in different media over 7 days.

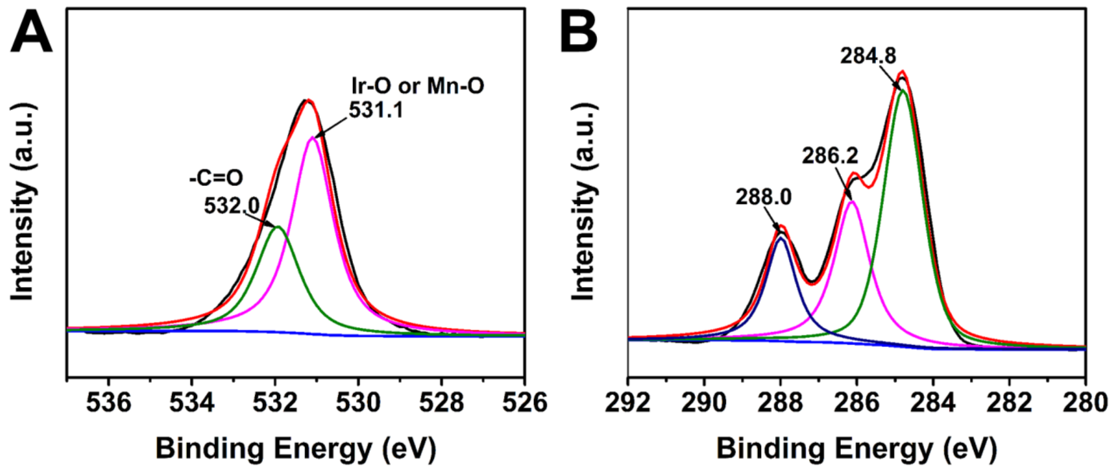


**Figure S5.** (A) O 1s and (B) C 1s XPS spectra of the BSA-Ce6@$IrO_2$/$MnO_2$ nanoparticles.

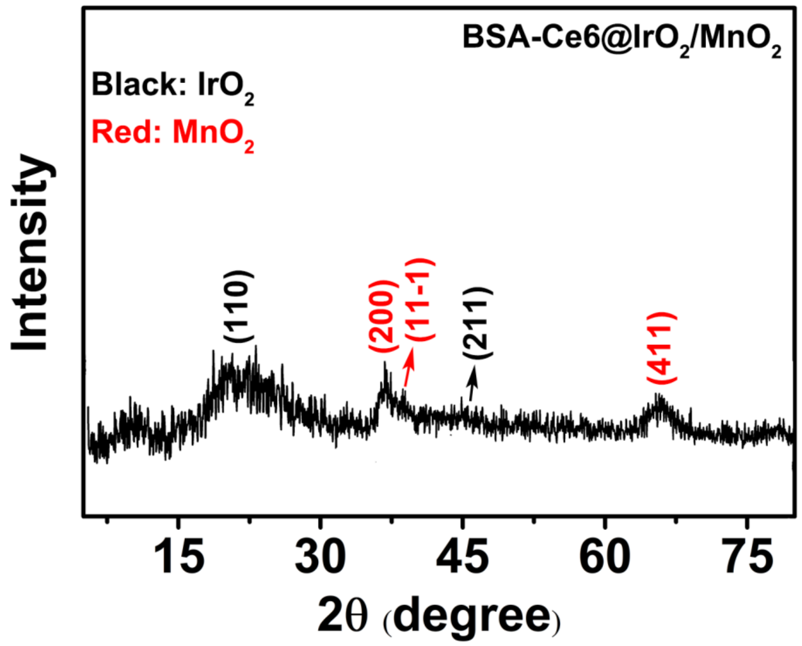


**Figure S6.** The X-ray diffraction pattern of BSA-Ce6@$IrO_2$/$MnO_2$ NPs.

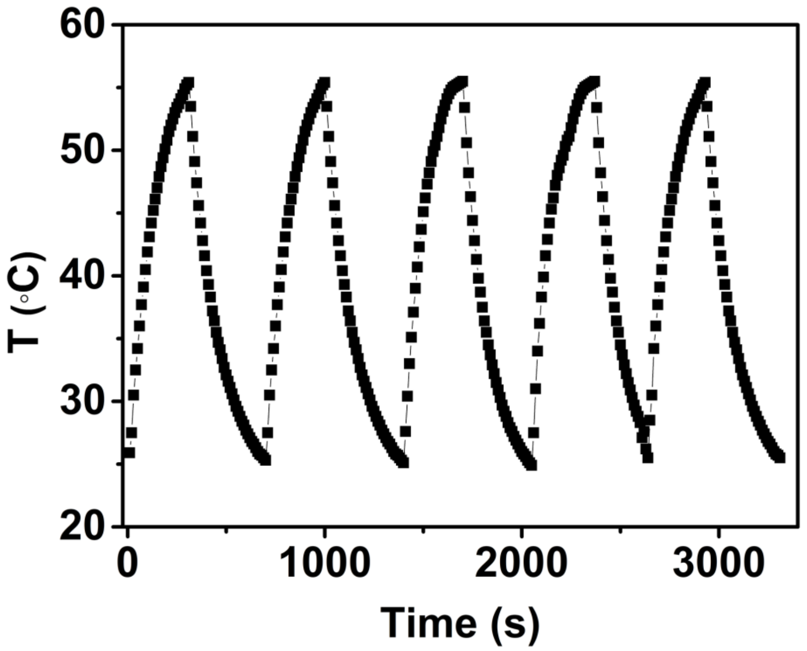


**Figure S7.** The temperature-time curve for a BSA-Ce6@$IrO_2$/$MnO_2$ dispersion (5 mM with respect to Ir) during five laser on/off cycles.

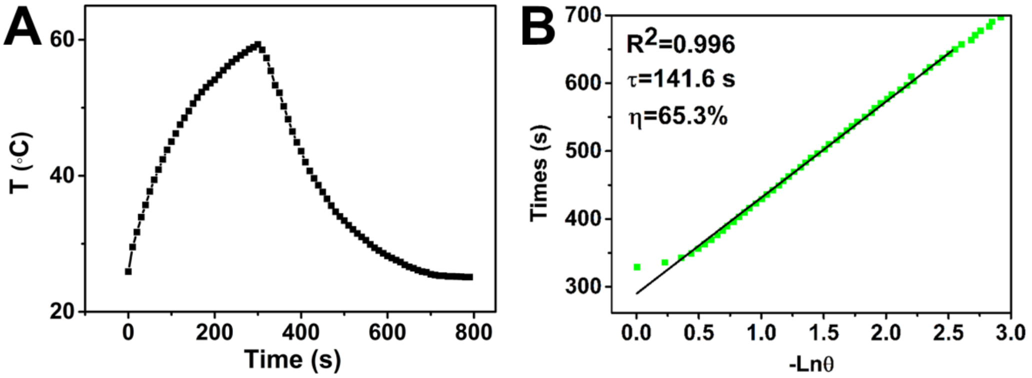


**Figure S8.** (A) Temperature change of a BSA-Ce6@$IrO_2$/$MnO_2$ dispersion (6.0 mM with respect to Ir) irradiated with an 808 nm laser. The laser was turned off after irradiation for 600 s. (B) The time constant for heat transfer of the BSA-Ce6@$IrO_2$/$MnO_2$ dispersion, calculated by plotting time data versus ln θ (defined as the ratio of $\Delta T$ to $\Delta T_{max}$) during the cooling process.

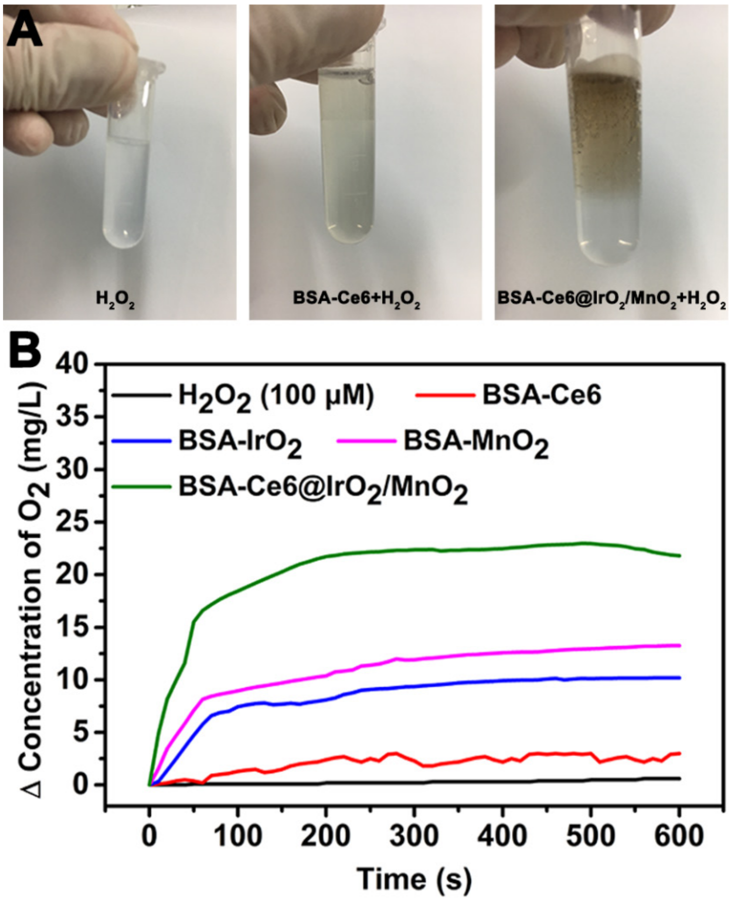


**Figure S9. (A)** Photographs of $H_2O_2$ solutions after incubation with BSA-Ce6 or BSA-Ce6@$IrO_2$/$MnO_2$ nanoparticles (Ir: 3 mM, Mn: 2 mM). (B) Oxygen generation in $H_2O_2$ solutions after incubation with BSA-Ce6, BSA-$IrO_2$ (Ir: 3 mM), BSA-$MnO_2$ (Mn: 2 mM) or BSA-Ce6@$IrO_2$/$MnO_2$ nanoparticles (Ir: 3 mM, Mn: 2 mM).

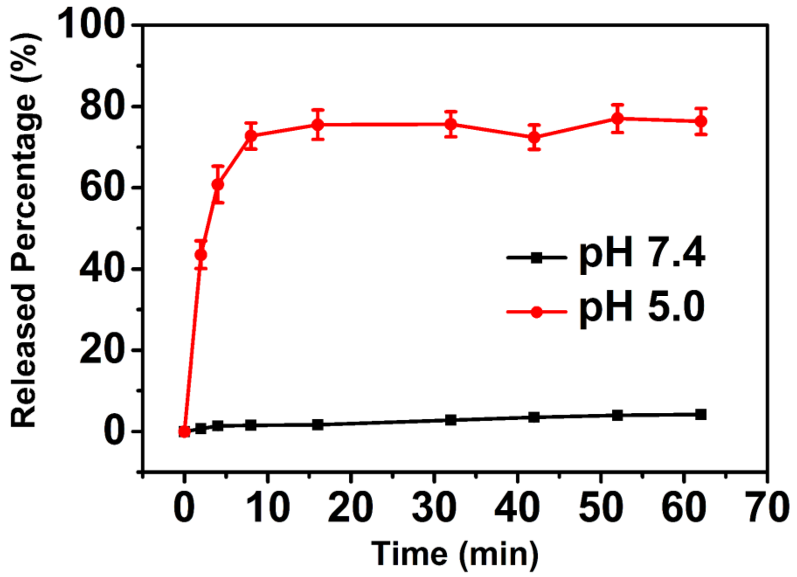


**Figure S10.** The percentage of $Mn^{2+}$ released from BSA-Ce6@$IrO_2$/$MnO_2$ NPs over time in PBS at different pH values (7.4, and 5.0). Results are shown as mean ± S.D. from 6 independent experiments.

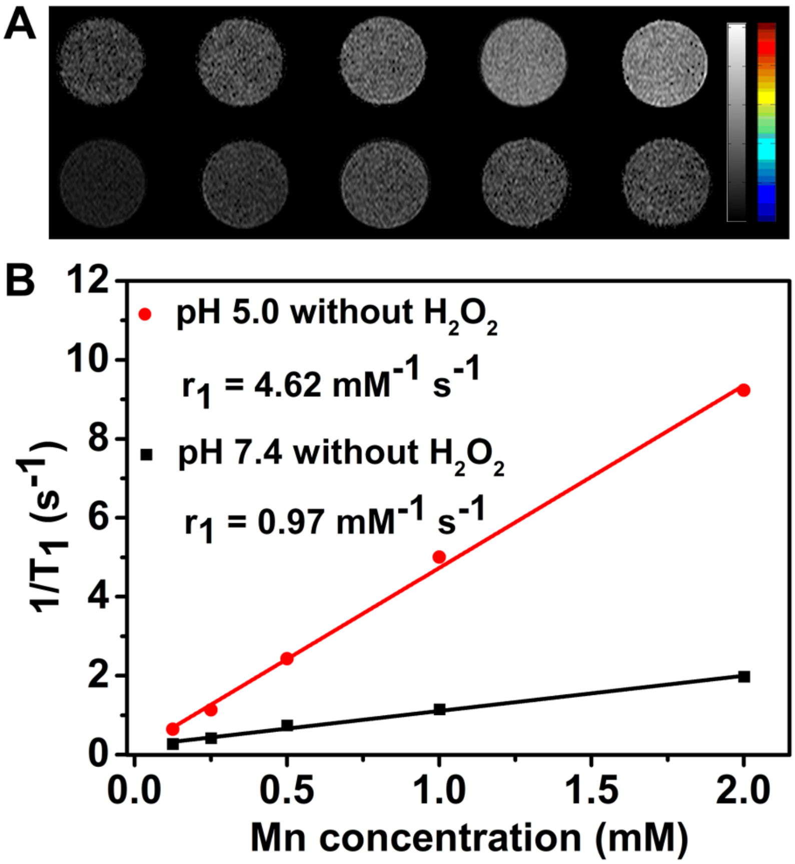


**Figure S11.** $T_1$-weighted MR images and relaxivity fits of BSA-Ce6@$IrO_2$/$MnO_2$ dispersions at pH 5.0 and 7.4 without $H_2O_2$.

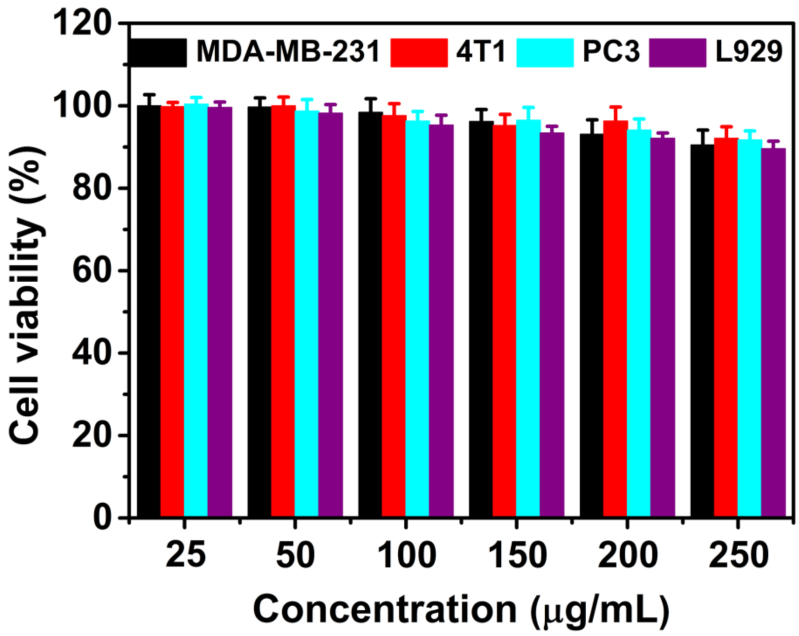


**Figure S12.** Relative cell viability of MDA-MB-231, 4T1, PC3, and L929 cells after incubation with different concentrations of BSA-Ce6@$IrO_2$/$MnO_2$ for 24 h. Values are given as a percentage relative to an untreated cells control (n = 5).

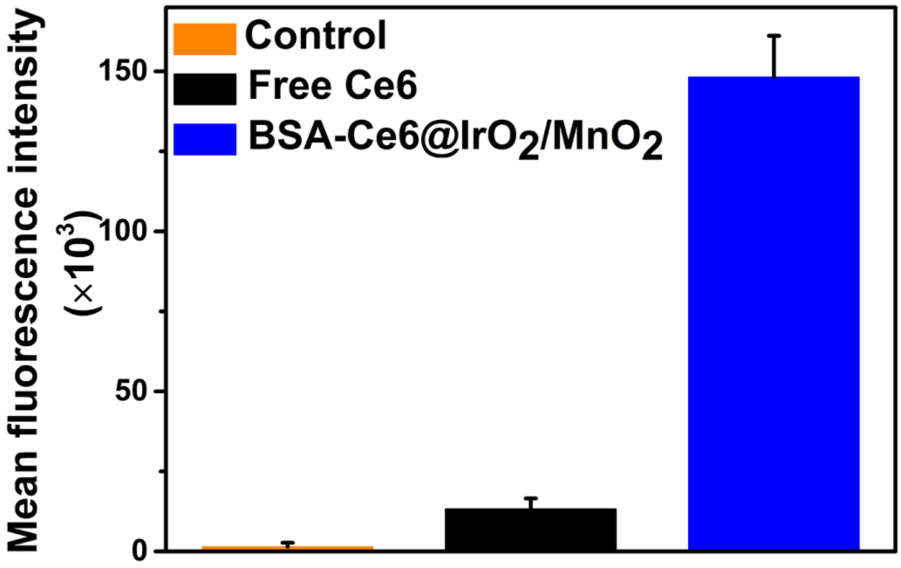


**Figure S13.** Quantitative mean fluorescence intensities derived from the flow cytometric analysis in Figure 4D (n = 5).

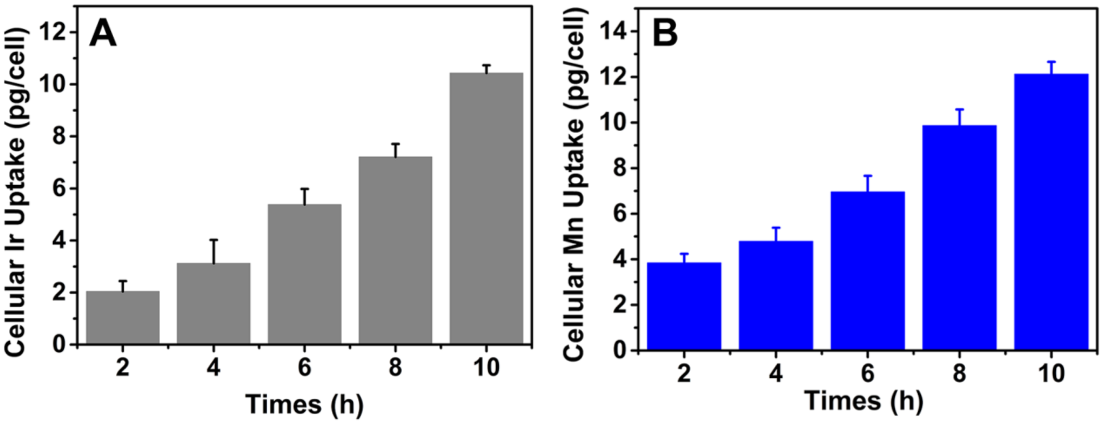


**Figure S14.** The uptake of (A) Ir and (B) Mn by MDA-MB-231 cells at different times after treatment with the BSA-Ce6@$IrO_2$/$MnO_2$ nanoparticles (n = 5).

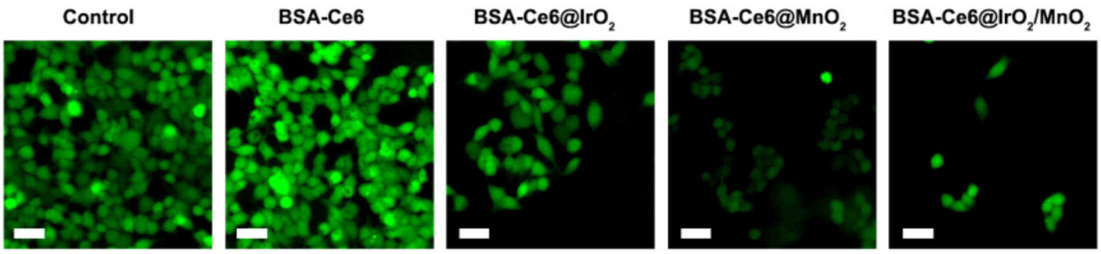


**Figure S15.** Intracellular $O_2$ production (as quantified using the RDPP $O_2$ probe) after MDA-MB-231 cells were incubated with different formulations (Ir: 3 mM, Mn: 2 mM). Scale bars: 50 μm.

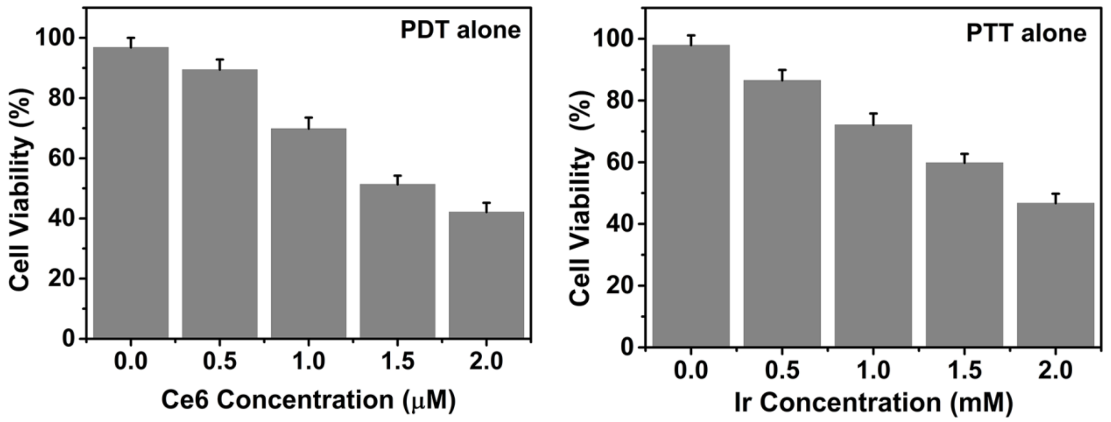


**Figure S16.** Concentration-dependent cell viability of MDA-MB-231 cells treated with BSA-Ce6@$IrO_2$/$MnO_2$. (A) PDT: 660 nm light irradiation (5 mW $cm^{-2}$, 30 min); (B) PTT: 808 nm (1.0 W $cm^{-2}$, 10 min).

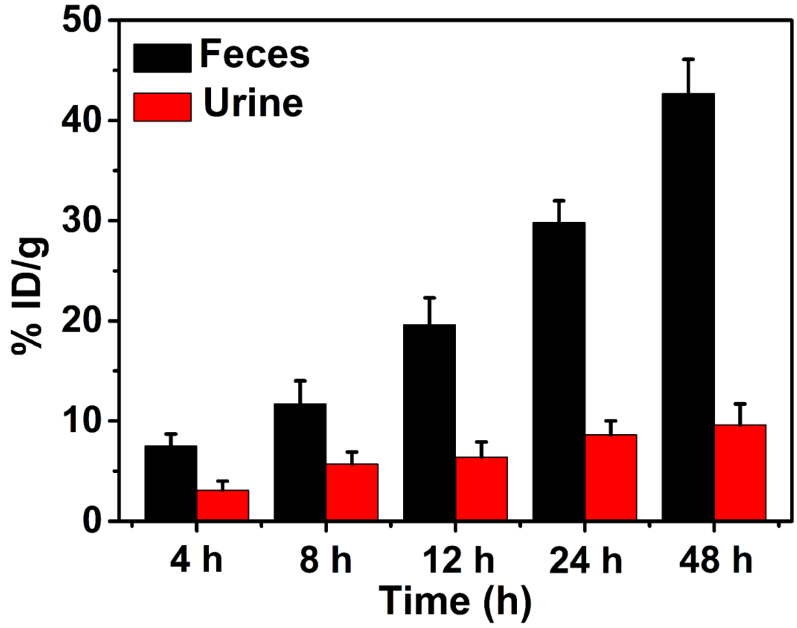


**Figure S17.** Quantification of BSA-Ce6@$IrO_2$/$MnO_2$ nanoparticles in urine and feces of MDA-MB-231 tumor-bearing mice at various time points after injection (n = 4).

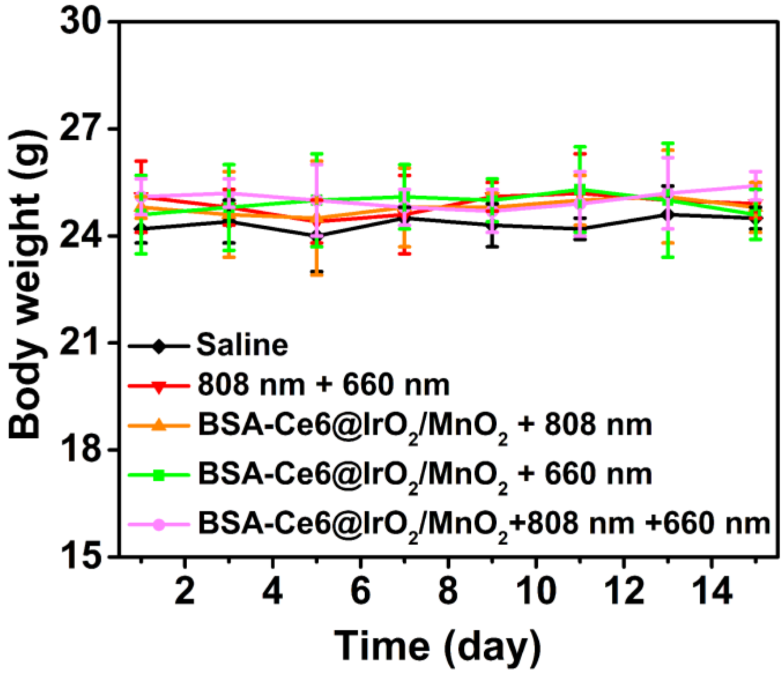


**Figure S18.** Body weight of MDA-MB-231 tumor-bearing mice during different treatments (n = 4).

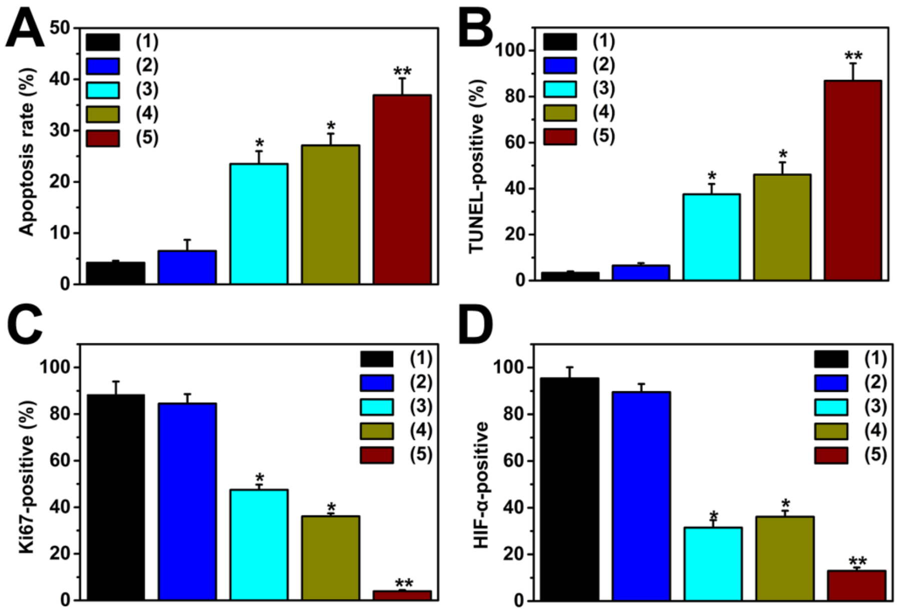


**Figure S19.** Quantitative analysis of (a) cell apoptosis, (b) TUNEL, (c) Ki67 and (d) HIF-α positive rates after the different *in vivo* treatments. **P < 0.01 and *P < 0.05 by Student's two-tailed t test. Groups are (1) saline, (2) 808 nm + 660 nm laser irradiation, (3) BSA-Ce6@$IrO_2$/$MnO_2$ + 808 nm laser, (4) BSA-Ce6@$IrO_2$/$MnO_2$ + 660 nm laser, and (5) BSA-Ce6@$IrO_2$/$MnO_2$ + 808 nm + 660 nm lasers, respectively.

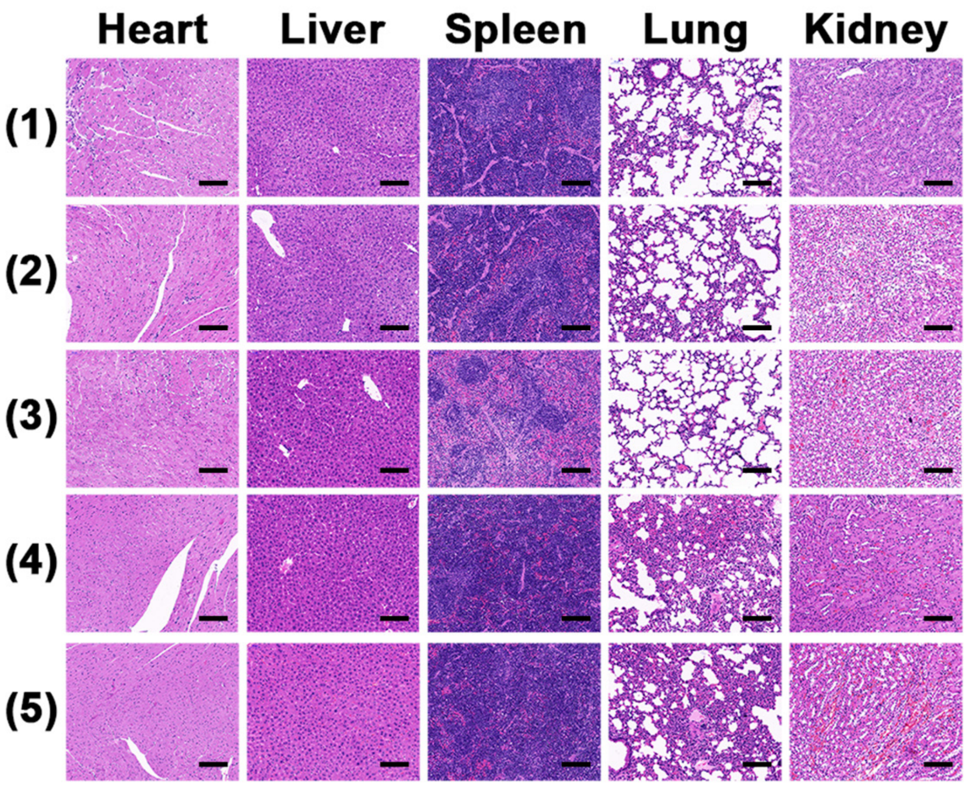


**Figure S20.** H&E-stained tissue sections of the major organs (heart, liver, spleen, lung, and kidney). Data were collected on day 15 from mice treated with (1) saline, (2) 808 nm + 660 nm laser irradiation, (3) BSA-Ce6@$IrO_2$/$MnO_2$ + 808 nm laser, (4) BSA-Ce6@$IrO_2$/$MnO_2$ + 660 nm laser, and (5) BSA-Ce6@$IrO_2$/$MnO_2$ + 808 nm + 660 nm lasers, respectively. Scale bars: 50 μm.

.

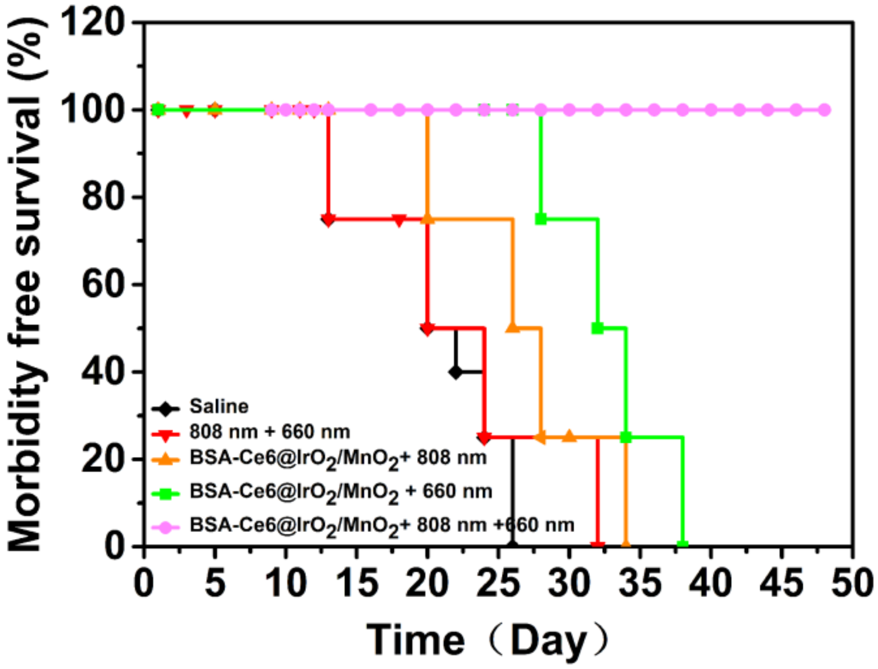


**Figure S21.** Survival rates of MDA-MB-231 tumor–bearing mice as a function of time post treatment.

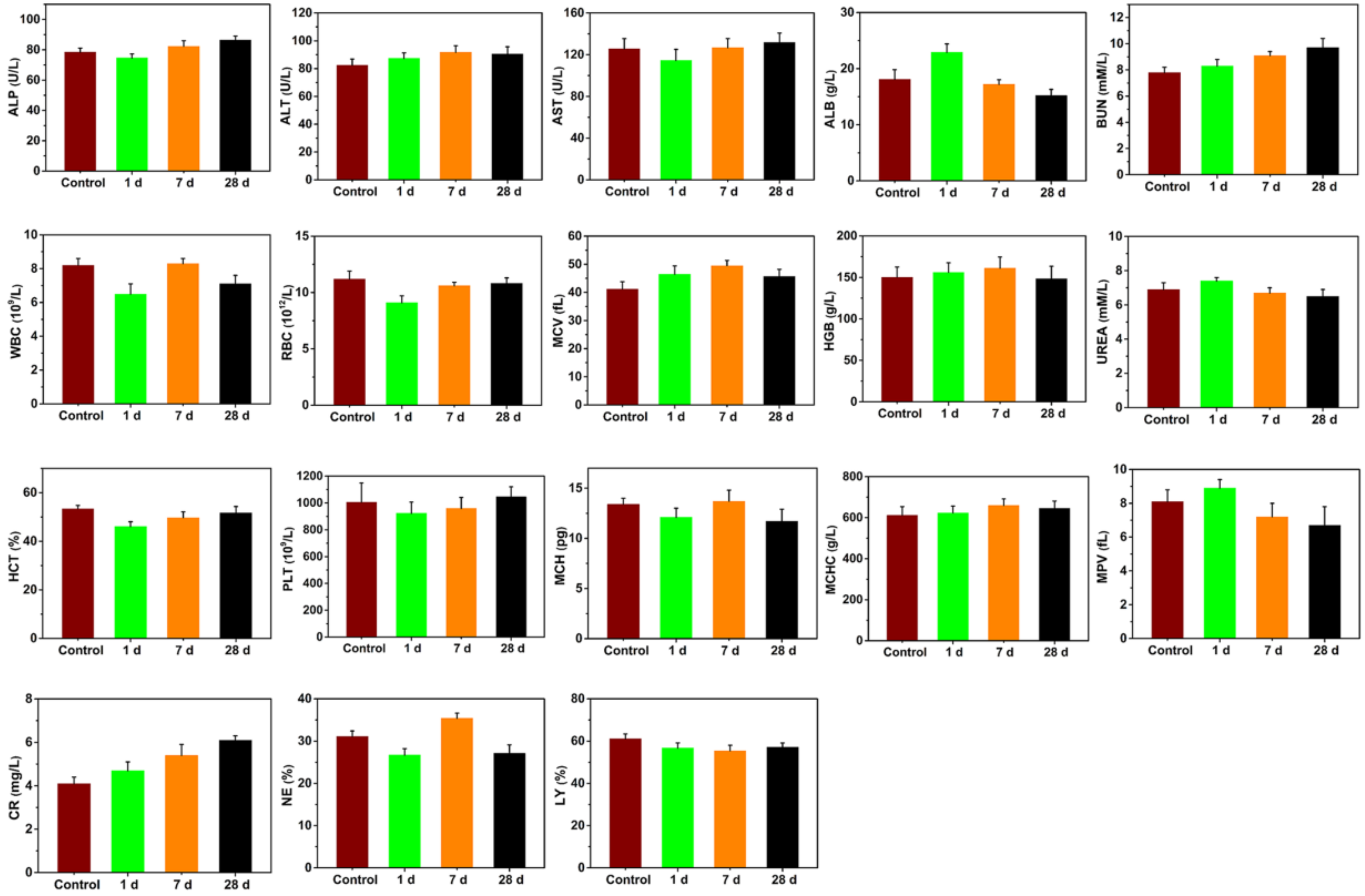


**Figure S22.** Blood biochemistry and hematology data of Balb/c mice treated with BSA-Ce6@$IrO_2$/$MnO_2$ nanoparticles at day 1, 7, and 28 after initial injection (n =4). The control samples are those taken at day 0 before treatment began.

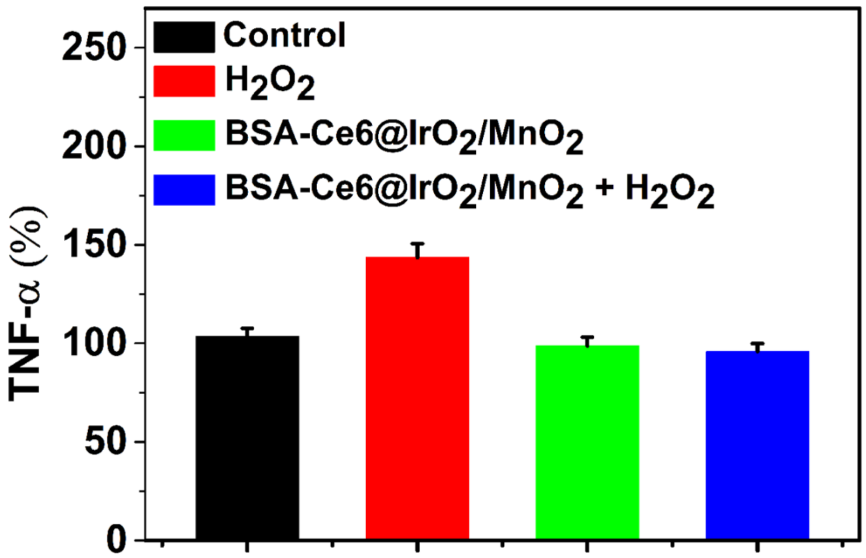


**Figure S23.** The TNF-α level in the sera of MDA-MB-231tumor-bearing mice after different treatments (n = 5).
